# Bis(2,2,2-trifluoroethyl) Carbonate As a Fire Suppressant Candidate for Lithium-Ion Batteries

**DOI:** 10.1021/acs.energyfuels.4c05359

**Published:** 2025-01-22

**Authors:** Maryam Khan-Ghauri, Pascal Diévart, Claire M. Grégoire, Keisuke Kanayama, Yousef Almarzooq, Shintaro Takahashi, Takuya Tezuka, Hisashi Nakamura, Laurent Catoire, Kaoru Maruta, Eric L. Petersen, Olivier Mathieu

**Affiliations:** † J. Mike Walker ’66 Department of Mechanical Engineering, Texas A&M University, College Station, Texas 77843, United States; ‡ CNRS-INSIS, I.C.A.R.E., 1C, Avenue de la Recherche Scientifique, 45071 Orléans Cedex 2, France; § Unité de Chimie et des Procédés (UCP), ENSTA Paris, Institut Polytechnique de Paris, Palaiseau 91762, France; ∥ Institute of Fluid Science, Tohoku University, Sendai, Miyagi 980-8577, Japan; ⊥ Mechanical Engineering Department, King Saud University, P.O. Box 800, Riyadh 11421, Saudi Arabia

## Abstract

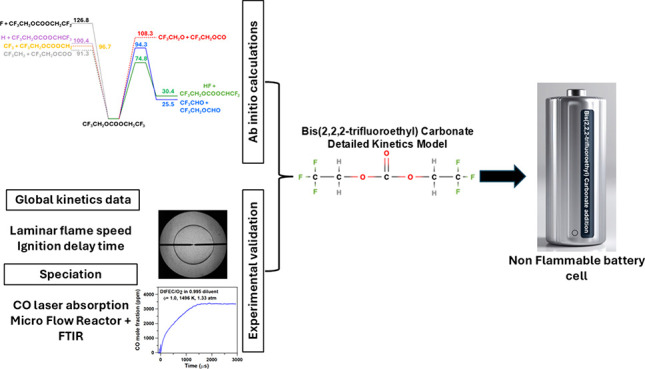

Bis(2,2,2-trifluoroethyl) carbonate (BtFEC) is a fire suppressant candidate for the use of lithium-ion batteries (LIBs). It is known that the electrolyte components in LIBs are highly flammable, making them susceptible to igniting, whether this is due to a manufacturing fault or an abuse of the LIB itself. To address this risk, the efficiency of BtFEC as a fire suppressant was investigated experimentally in a high-temperature combustion environment, allowing for further refinement and validation of the model. Using a shock tube, BtFEC combustion properties were measured experimentally behind a reflected shock wave, capturing OH* chemiluminescence to assess ignition delay times (IDT) as well as CO time-history profiles through the implementation of laser absorption spectroscopy. Both pyrolysis and oxidation conditions were captured with three equivalence ratios (ϕ = 0.5, 1.0, and 1.5) for a temperature range of ∼1200–1650 K at near-atmospheric pressures. In addition, key species measurements were taken using a microflow reactor (MFR) with a controlled temperature profile associated with Fourier transform infrared spectroscopy (FTIR). Key species investigated were BtFEC, CO, CO_2_, CHF_3_, CF_2_O, C_2_F_6_, and HF for the temperatures range of 800–1300 K. MFR measurements allowed for a new set of measurements by which to validate the model compared to the previous study [Mathieu et al. *Proc. Combust. Inst.***2023**, *39*, 499] where the first assembly of the model used CO time-history, IDT, and laminar flame speed measurements. Refinement of the model was carried out with new high-level calculations as well as sensitivity, rate-of-production, and reaction pathway analyses using recent reaction rate updates from the literature. The modifications led to improvements in the level of agreement between the kinetic modeling and the new experimental data.

## 1 Introduction

As the global demand for electric vehicles and portable devices has increased exponentially, so has the necessity for lithium-ion batteries (LIBs). LIBs’ widespread use has shone a light on the significant fire hazards associated with them,^1^ with this fire hazard being due to either defaults in the manufacturing processes or to abuse of usage. A major concern with the use of LIBs is the flammability of the electrolyte, a component of the battery important for the charge–discharge cycles, because it allows lithium salt ions to move between the electrodes. By understanding the reactions involved in this combustion process, the risk can potentially be reduced with the addition of a specific fire suppressant able to reduce this flammability. Phosphorus-based flame retardants such as trimethyl phosphate (TMP),^2−5^ triethyl phosphate (TEP),^6^ or triphenyl phosphate (TPP)^7,8^ are frequently considered as low-concentration additives to the LIB electrolyte due to the well-known flame-retardant properties of phosphorus.^9,10^

Other types of agents considered are fluorinated agents with structures similar to those of the LIB electrolyte components. The basic idea is that the F atoms trap radical H’s to form the stable HF molecule and prevent chain-branching and chain-propagating reactions, which are at the heart of the combustion process, from occurring, with the added benefit that the similar structure allows for a relatively seamless integration to the electrolyte. One can mention fluoroethylene carbonate (FEC)^11,12^ and difluoroethylene carbonate (DFEC).^13^ Both FEC and DFEC have a structure similar to that of ethylene carbonate (EC), and their addition tends to improve the formation of the solid-electrolyte interphase (SEI) on the anode, which enhances the thermal stability of the electrolyte and can help mitigate capacity loss during cycling.

**Figure 1:**
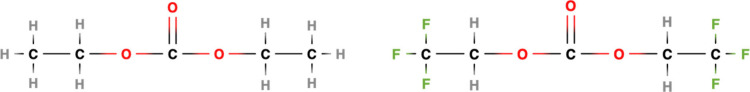
Molecular structure of diethyl carbonate (DEC; left) and bis(2,2,2-trifluoroethyl) carbonate (BtFEC; right).

Another candidate that may suit this role is the molecule bis(2,2,2-trifluoroethyl) carbonate (BtFEC), which has a similar structure to the linear carbonate diethyl carbonate (DEC), a common constituent of LIB liquid electrolytes.^14^ The structures of BtFEC and DEC are shown in Figure 1 for comparison. This comparable structure of BtFEC and DEC presents a chance to reduce the likelihood of battery-started fires without the addition of unwanted physical or chemical processes within the electrolyte or the electrodes, therefore not degrading the performance of the battery itself or even possibly improving it. For instance, it has been shown in recent studies that the use of fluorinated carbonates like BtFEC in battery electrolytes can be beneficial to high-voltage LIBs by improving high-temperature performance, preventing large oxidation of the electrolyte on the cathode surface, and preventing transition metal dissolution.^15−17^ These findings and the potential of BtFEC as a safety agent make the development of an accurate and detailed chemical kinetic mechanism for BtFEC oxidation important.

The only detailed mechanism available in the literature for BtFEC has been proposed in a collaborative effort, where ab initio calculations were used to determine the main reaction pathways and reaction rate coefficients for the BtFEC subset. This model was validated by CO profiles in pyrolysis conditions,^18^ and the fire suppressant effectiveness was also assessed by comparing well-known combustion properties, i.e., laminar flame speeds (LFS) and peak OH* ignition delay times (τ_OH*_) for well-studied fuels (H_2_ and CH_4_) seeded with a small amount of BtFEC. The aim of this study was to build on this work by updating the model using new ab initio computations and the recent literature progress, and to validate this mechanism using new experimental data. Below is a description of the experimental apparatuses used, followed by a detailed breakdown of the reactions added/updated by recent literature and new computations. The results of the model are shown in comparison to the experimental data for CO profiles under pyrolysis conditions as well as oxidation for highly diluted BtFEC/O_2_ mixtures. τ_OH*_ values for these mixtures are also presented alongside τ_OH*_ results for seeded mixtures of BtFEC/H_2_ and BtFEC/CH_4_ in comparison to the updated model. The seeded mixtures for a fixed percentage of BtFEC are also compared for results of LFS measurements from Mathieu et al.^18^ Results of the current model are then provided for Micro-Flow Reactor (MFR) data and are compared with experimentally obtained data to monitor a range of species concentrations (BtFEC, CO, CO_2_, CHF_3_, CF_2_O, C_2_F_6_, and HF) at different temperatures. A thorough analysis is then provided to highlight the chemistry underlying these results.

## 2 Methods

### 2.1 Experimental and Computational Methods

#### 2.1.1 Shock-Tube Facility

Shock-tube experiments were conducted at Texas A&M University using a stainless-steel apparatus with a driver section measuring 3.25 m in length and 7.62 cm in inner diameter, and a driven section measuring 7.88 m in length and 16.2 cm in inner diameter. The two sections are separated by a 0.25-mm-thick polycarbonate diaphragm to generate pressures of ∼1.3 atm behind the reflected shock wave. For the diaphragm rupture to be consistent, a cross-shaped cutter is placed down from the diaphragm, giving an ideal rupture for each experiment. The incident shock-wave velocity was measured using five piezoelectric pressure transducers to detect the shock passing toward the end section of the shock tube. Through this method of shock detection, alongside the use of the normal shock equations, the temperature (*T*_5_) and pressure (*P*_5_) behind the reflected shock wave can be calculated within an uncertainty of ±1% and ±0.8%, respectively.^19^ The mixture is highly diluted with argon, ensuring minimal boundary-layer effects^20^ and minimizing temperature changes due to chemical reactions. Another component of the diluent is 20% helium, which is used to accelerate the vibrational relaxation of CO, enabling high-sensitivity laser absorption diagnostics to be used for model validation, see Mathieu et al.^21^ The use of helium as a driver gas and the specific geometry of the shock tube allow test times behind the reflected shock wave of up to 3 ms, during which chemical kinetics measurements can be taken under nearly ideal conditions. To obtain a suitable vacuum prior to each experiment, a turbomolecular pump is used, backed by a rotary-vane pump to ensure a pressure of 10^–8^ Torr. Details on this process can be found in Cooper et al.^22^

#### 2.1.2 Chemiluminescence Emission and CO Laser Absorption Diagnostics

**Figure 2:**
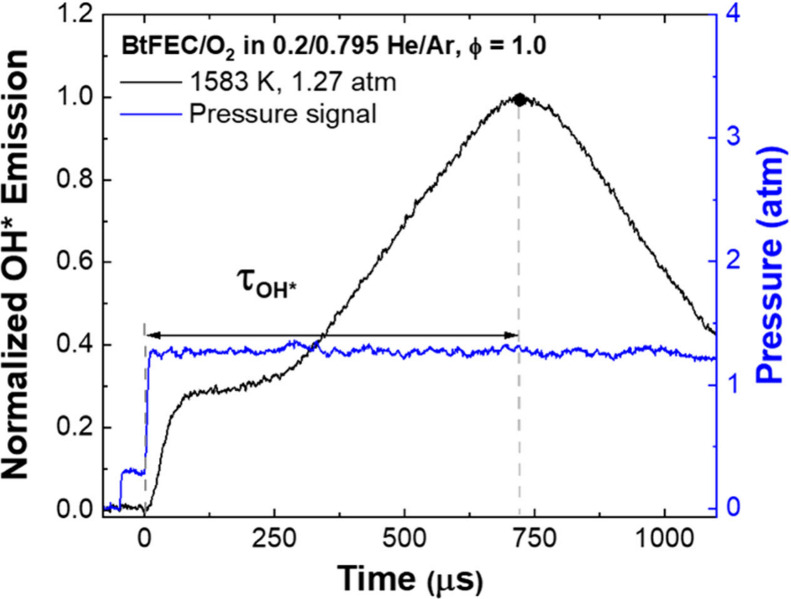
Illustration of the peak OH* time using the experimental OH* and pressure profiles for the oxidation of BtFEC in 0.2/0.795 He/Ar, ϕ = 1.0, at 1583 K and 1.27 atm.

The chemiluminescence from the de-excitation of the radical species OH* was followed using an interference filter centered at 307 ± 10 nm. This wavelength corresponds to the *A*^2^∑^+^ → *X*^2^Π transition, and the emission is collected using a photomultiplier tube in an in-house casing. Peak OH* ignition delay times (τ_OH*_) can be determined from time-history profiles of OH* as seen in Figure 2. τ_OH*_ was defined as the time between the pressure rise due to the reflected shock detected at the sidewall and the maximum peak of OH* production. This method leads to an uncertainty of ±15% in the τ_OH*_.

To measure CO time histories, a tunable quantum cascade laser produced light near 4.8 μm to monitor the P^20^ line of the 1 ← 0 CO band. Prior to each experiment, the CO wavelength was centered to the peak of the P^20^ line at conditions of 30 °C and ∼196 mA. A removable cell containing the mixture ∼10% CO/90% Ar is used to verify the maximum absorption strength and allows for fine-tuning to find this maximum. Due to the wavelength used, this technique works for CO with relative isolation from H_2_O and CO_2_ absorption.^23^ To find the CO time histories, the Beer–Lambert Law is implemented by using the relation of the same name in eq 1.
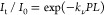
1with *k*_*v*_ the absorption coefficient, *P* the partial pressure, and *L* the path length (inner diameter of the shock tube). *I*_t_/*I*_0_ is found experimentally as the optical layout splits the laser beam into two components: the time-resolved incident intensity (*I*_0_) and the time-resolved transmitted intensity (*I*_t_). The absorption coefficient is calibrated for the temperature range 1180 to 2190 K using a mixture of 2000 ppm of CO in 0.2 He/0.798 Ar. The temperature-dependent equation found below can be used to describe *k*_ν_ (cm^–1^ atm^–1^) with an *R*^2^ value of 0.9984. This method of calibration was recently validated by Grégoire et al.^24^ with the use of CO spectroscopy.
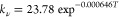
2

**Figure 3:**
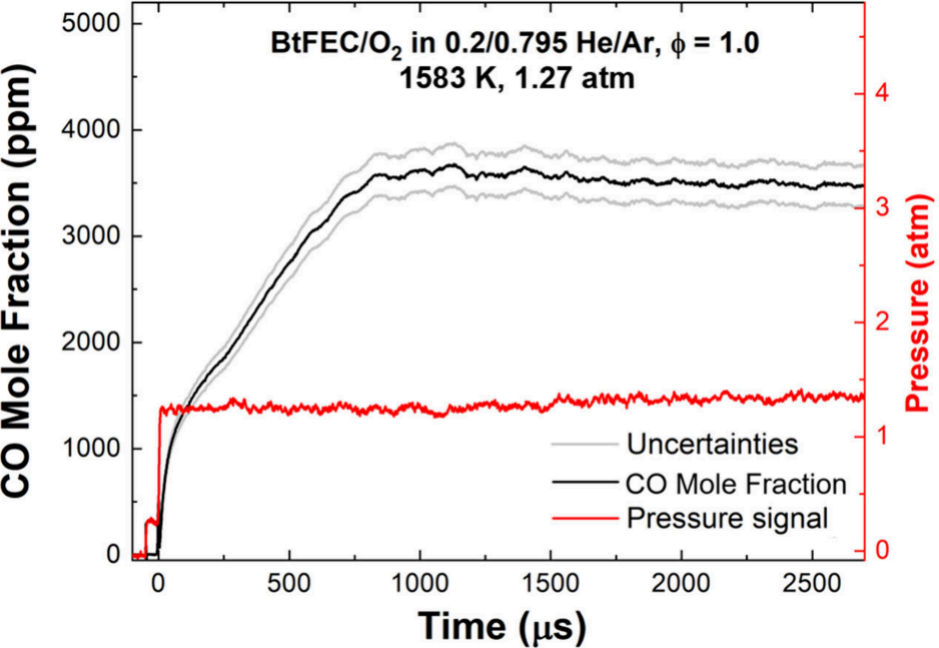
Illustration of the CO time-history profile uncertainty for the oxidation of BtFEC in 0.2/0.795 He/Ar, ϕ = 1.0, at 1583 K and 1.27 atm.

During each experiment, a 40–90 K increase in temperature can be expected due to oxidation reactions. The exact increase within the range is contingent on the experimental conditions. This small adjustment was accounted for in eq 2 using computed time-varying temperature with the model and therefore creating an absorption coefficient that is also time-varying. The uncertainty in CO concentration is estimated to be within ±5.5%,^25^ and an example of the effect of this uncertainty on a representative CO profile is visible in Figure 3.

During the experiments, both OH* and CO were measured simultaneously over the temperature range 1240 to 1624 K at near-atmospheric pressure (1.25–1.42 atm) covering three equivalence ratios ϕ = 0.5, 1.0, and 1.5. Mixture compositions in relation to each equivalence ratio and experimental conditions are detailed in Table 1, with the equivalence ratio determination based on the overall combustion reaction of BtFEC (reaction R1, with the reaction numbers corresponding to their appearance order, as they are discussed herein).
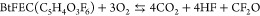
R1

**Table 1:** Mixture Compositions and Experimental Conditions Covered during Shock-Tube Measurements for OH* and CO

ϕ	*X*_BtFEC_	*X*_O2_	*X*_He_	*X*_Ar_	temperature (K)	pressure (atm)
0.5	0.00071	0.00429	0.2	0.795	1288–1609	1.25–1.42
1.0	0.00125	0.00375	0.2	0.795	1240–1583	1.27–1.41
1.5	0.00167	0.00333	0.2	0.795	1265–1624	1.25–1.39

Note that the products in reaction R1 were determined using equilibrium calculations with Ansys Chemkin. This step was necessary because, while the final product for F atoms in systems containing H atoms during combustion is HF, BtFEC contains more F atoms than H atoms. The equilibrium calculation showed that CF_2_O was greatly favored for F-containing products other than HF under our conditions and was used to balance the equation.

#### 2.1.3 Microflow Reactor with a Controlled Temperature Profile

**Figure 4:**
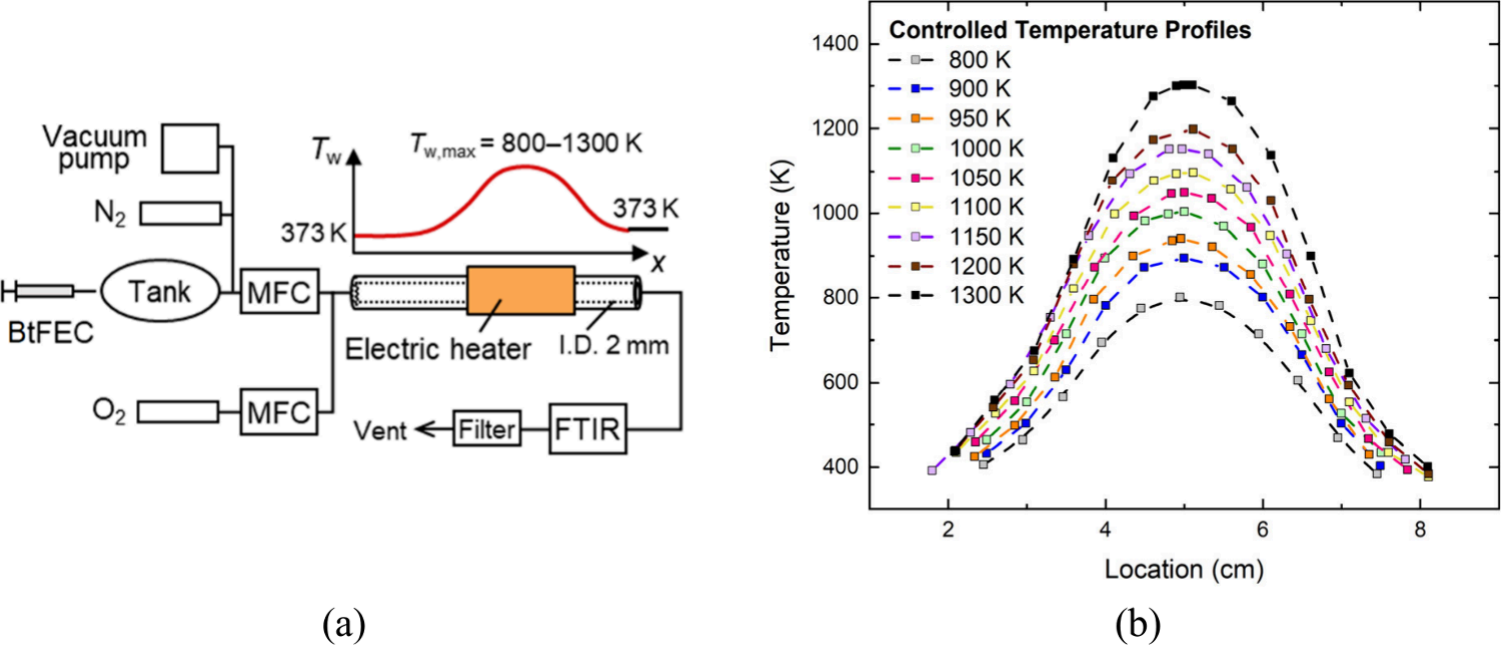
(a) Schematic of the MFR experimental setup and (b) measured temperature profiles.

Species measurements for BtFEC pyrolysis and oxidation were conducted at Tohoku University using a microflow reactor with a controlled temperature profile (MFR)^26^ and Fourier-transform infrared spectroscopy (FTIR). Details on the experimental method can be found in refs (27) and (28). To summarize, a quartz tube with an inner diameter of 2 mm heated with an electric heater was used as the reactor channel. Stationary temperature profiles are formed on the inner wall surface of the reactor, as the gas-phase temperature profile is strongly governed by the given wall temperature profile due to a small Peclet number within the MFR. The maximum wall temperatures were varied between 800 and 1300 K, and the temperature profiles were measured using K-type thermocouples before the experiments. A schematic of the MFR experimental setup is presented in Figure 4, along with the measured wall temperature profiles with an uncertainty estimated to be ±5 K.

BtFEC/N_2_ mixtures were prepared in a heated tank by a partial pressure method. The BtFEC/N_2_ mixtures were supplied to the reactor with and without O_2_ at equivalence ratios of 0.5, 1.0, and 1.5 for the oxidation and pyrolysis conditions with an inlet mean flow velocity of 2.0 cm/s and atmospheric pressure. The exhaust gases were directly introduced into a gas cell whose window was made of BaF_2_ that allows excellent transmission from the deep ultraviolet to the far-infrared. The FTIR spectra were taken with 0.25 cm^–1^ nominal resolution by co-adding 30 scans and were analyzed in the mid-IR wavenumber region from 750 to 4000 cm^–1^. The absorption peaks were chosen based on an IR spectrum library in the NIST database and preliminary experiments to achieve high detection sensitivities (details provided in the Supporting Information) with minimal interference from other species in the reaction mixture.^28^ The present study identified BtFEC (1329.39 cm^–1^), CO (2183.35 cm^–1^), CO_2_ (2360.31 cm^–1^), CF_2_O (1929.00 cm^–1^), CHF_3_ (1154.60 cm^–1^), C_2_F_6_ (1114.34 cm^–1^), and HF (3878.00 cm^–1^). All species were quantified as well, except HF (a safety measure to not have a high-concentration HF tank in the laboratory for otherwise necessary calibration). The lines from the heated tank to the reactor and the sampling line were kept at 373 K to prevent condensation of unburned BtFEC and water vapor. HF in the vent gas was removed through a chemical filter of calcium hydroxide (Ca(OH)_2_). Table 2 summarizes the mixture compositions during the MFR experiments.

**Table 2:** Mixture Compositions and Temperature Conditions Used during the MFR Experiments

ϕ	*X*_BtFEC_	*X*_O2_	*X*_N2_	temperature (K)
0.5	0.015	0.090	0.895	800–1300
1.0	0.015	0.045	0.940	800–1300
1.5	0.015	0.030	0.955	800–1300
∞	0.015	0	0.985	800–1200

The flow field in the MFR at low flow velocities can be modeled as a one-dimensional, steady-state reactive flow. The Cantera-based code with a convective heat transfer term between the gas and the reactor wall added in the energy equation was^29^ used in this study. The same conditions for the experiments (mixture compositions, inlet flow velocity, pressure, and wall temperature profiles) were applied to the computations. The measured wall temperature profiles and those used in the computation can be found in ref (27). The computational domain was set to 0–10 cm, and mole fractions at the end of the computational domain were compared with the measured data. Measured and computed MFR data and wall temperature profiles used in computations are available in the Supporting Information.

### 2.2 Detailed Chemical Kinetics Modeling

#### 2.2.1 Theoretical Methods

The decomposition of BtFEC has been reinvestigated theoretically. The density functional M06-2x-D3 as implemented in Gaussian 09^30^ was used alongside the triple-ζ basis set def2-TZVPD to determine the geometries and the harmonic vibrational frequencies of stable and transient structures. The optimized geometries were verified to be the lowest energy conformers by performing M06-2x/def2-SVPD relaxed scans (by 15° steps) around all of the hindered dihedrals. A series of single point energies were then performed in Orca^31^ with the DLPNO–CCSD(T) and CCSD(T) methods and the Dunning’s basis sets of increasing size (D, T, Q) within the frozen-core approximation. Hartree–Fock and correlation energies were then extrapolated separately to the infinite basis set limit using a mixed exponential/Gaussian scheme (*E*(*n*) = *E*_∞_ + *Ae*^–(*l*–1)^ + *B*_*e*_^–(*l*–1)^2^^).

For the correlation energy, the second-order coefficient was derived from the DLPNO–CCSD(T) energies since the cost of the CCSD(T)/cc-pVQZ calculation was prohibitive for the large species investigated herein. The interaction of the valence and core electrons was taken into account by introducing a correction term *E*_CV_, which is defined as the difference between the frozen-core and the full-potential DLPNO–CCSD(T)/cc-pCVTZ energies. The final energies at 0 K reported hereafter are the summation of the three contributions aforementioned and the harmonic zero-point vibrational energy (ZPVE) scaled by 0.976.^32^
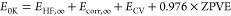
3

For the reactions involving smaller species (up to two carbon atoms), a similar procedure was adopted, however, with the B2PLYP method instead of the M06-2x for geometry optimization and frequencies calculations.

#### 2.2.2 Rate Constant Calculations

**Figure 5:**
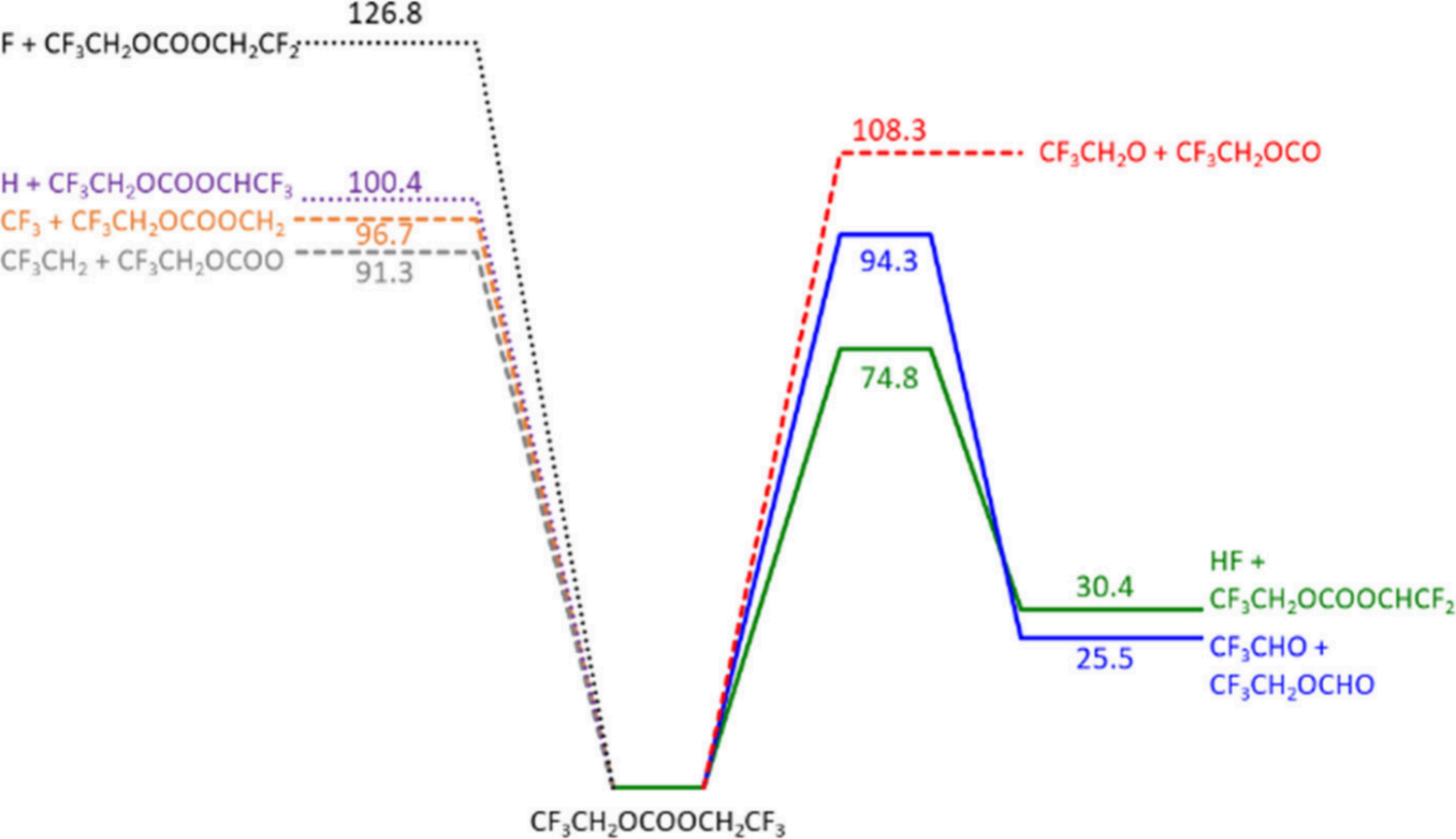
Schematic of the potential energy surface for the BtFEC thermal decomposition. Energies (kcal mol^–1^) are relative to the fuel.

Regarding the decomposition of BtFEC, a ME/RRKM analysis was performed based on the potential energy surface (PES) displayed in Figure 5 and microscopic properties (geometries, harmonic frequencies scaled by 0.9876,^32^ hindered rotors PES) of the reactant, products, and saddle points from the aforementioned theoretical calculations. For the barrierless reactions, a variational analysis was performed using the semiempirical phase-space theory (PST) method implemented in the master equation system solver (MESS). The coefficients of the interaction potential for each reaction were adjusted in order to recover the high-pressure limit for the radical–radical recombination (Table 3). Asymmetric Eckart tunneling was included for reactions involving a true saddle point. A single exponential model (⟨Δ*E*_down_⟩ = 400(*T*/300)^0.85^) was adopted for the collisional energy transfer and Lennard-Jones parameters ϵ/*k* = 380.4 K and σ = 7.05 Å of the initial fuel were calculated from corresponding state theory.

**Table 3:** Recombination Rate Constants Employed in the ME/RRKM Analysis

reaction	rate constant [cm^3^ s^–1^]	
		analogy to the reaction CH_3_ + C_2_H_5_^33^
		from the room temperature rate constant of CH_3_ + CH_3_OCO^34^ and the temperature dependence of CH_3_O + CH_3_^35^
		analogy to the reaction CH_3_ + CH_3_CO^36^
		analogy to the reaction H + iC_3_H_7_^37^

The rate constants of bimolecular reactions, for which there is no pressure-dependence, was evaluated with conventional transition state theory (CTST) with asymmetric Eckart tunnelling and Pitzer-Gwinn corrections for hindered rotors as implemented in the module *Thermo of MultiWell*.^38^

#### 2.2.3 Ab Initio Calculation Results

Diethyl carbonate mainly decomposes through a concerted molecular elimination,^39^ in which an H atom of a methyl group is transferred to an O atom; however, such paths do not exist for BtFEC. Nonetheless, H atom transfer can still occur but through tighter concerted elimination, resulting in higher energy barriers. Two elimination pathways were identified, leading to the elimination of either an HF or a trifluoroacetaldehyde molecule through two distinct four-membered ring transition states, with associated energy barriers of 74.8 and 94.3 kcal mol^–1^, respectively. BtFEC decomposition can also proceed through homolytic bond scissions with energy barriers ranging from 91.3 to 128.7 kcal mol^–1^. It is noteworthy that the energy calculated here does not differ much from those in our previous study.

Pressure- and temperature-dependent rate constants for each BtFEC decomposition evaluated by the ME/RRKM analysis were fitted into the sum of two non-Arrhenius expressions in order to be implemented in the detailed kinetics model. This analysis reveals that at temperatures below 900 K, BtFEC mostly decomposes through the concerted HF elimination channel while the breaking of the C–O bond is dominant above 1000 K. The resulting rate constants are shown in Table 4.

**Table 4:** Rate Constant Coefficients of BtFEC (CF_3_CH_2_OCOOCH_2_CF_3_ in the Model) Pyrolysis Reactions^a^

reactions (updated)	*A*	*n*	*E*_a_
	2.65 × 10^70^	*–16.07*	115225
	**5.81 × 10**^**30**^	**–5.16**	**81557**
	**8.16 × 10**^**90**^	**–21.40**	**135260**
	2.50 × 10^85^	*–19.73*	136786
	**3.85 × 10**^**54**^	**–11.55**	**108805**
	**2.07 × 10**^**114**^	**–27.47**	**163534**
	3.67 × 10^80^	*–18.45*	127136
	**1.36 × 10**^**44**^	**-8.26**	**98835**
	**4.54 × 10**^**94**^	**–21.96**	**141460**
	3.50 × 10^96^	*–23.06*	153663
	**6.08 × 10**^**116**^	**–28.29**	**176195**
	**4.67 × 10**^**106**^	**–27.92**	**139292**
	3.59 × 10^90^	*–21.98*	150931
	**7.45 × 10**^**112**^	**–27.04**	**171941**
	**3.84 × 10**^**90**^	**–22.98**	**129332**
	**5.43 × 10**^**37**^	**–7.57**	**103192**
	**2.03 × 10**^**113**^	**–28.02**	**168635**

a In italic are the reaction rate coefficients used in Mathieu et al.,^18^ while the ones in bold correspond to the updated rate constants (sum of two non-Arrhenius expressions). In the updated reaction rate, a PLOG formalism is used; however, the rate coefficients reported are for 1 atm. Note that (*k* = *AT*^*n*^ exp(−*E*_a_)/*RT*)), units in cal, mol, K, and s.

#### 2.2.4 Model Improvements

Chemical reactions for BtFEC are based on previous work from our group,^18^ and the following modifications to improve said model were applied:

(1) BtFEC pyrolysis reactions were updated using *ab initio* calculations from the authors, as described in the previous section and shown in Table 4.
(2) The flame propagation for CH_2_F_2_/O_2_/N_2_ mixtures was studied by Burgess et al., and a chemical kinetics mechanism was produced.^40^ From this study, 26 reaction rates were updated in our model, and four new reactions were added, with a focus on the species CH_2_F_2_, CHF, and CF_2_. The thermochemistry and transport data for new radicals involved in these reactions, namely, FO, HOF, F_2_O, and F_2_, were added to the base chemistry of the BtFEC model (Table S1 in the Supporting Information).
(3) Sharma et al. studied abstraction reactions for fluoromethane combustion.^41^ A total of 39 reactions were calculated using *ab initio* electronic theory in which 33 were added to the current mechanism, five were updated using the newly calculated rate constants, and the duplicated reaction H + CF_2_O ⇆ HF + CFO was replaced with one unique reaction (Table S2 in the Supporting Information).
(4) Babushok et al. studied the inhibition of a premixed methane-air flame by 2-BTP (2-bromo-3,3,3-trifluoropropene)^42^ providing new rate constants for 26 H/F/C reactions. Of these, 18 were added to the mechanism, and three reactions involving the molecules CF_3_COF, CF_3_CF_3_, and CF_3_CO were updated using the rate constants provided in the study. Three reactions were not updated using rate constants from ref (42) as they had been updated with more recent rate constants from Sharma et al.^41^ already (Table S3 in the Supporting Information).

After implementation of the updates above based on recent publications, the model was further refined. Of particular interest for CO time-history profiles was reaction R2, shown below. As this reaction was found to be of high importance in producing CO, an *ab initio* calculation was carried out as described previously, giving reaction rate coefficients visible in Table 5. HCO is an important precursor to the production of CO, as described below, meaning that this alteration allowed for a much improved pyrolysis profile.
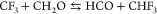
R2

**Table 5:** Rate Constant Coefficients of Updated Reactions Found to Improve the Current Model^a^

reactions (updated)	*A*	*n*	*E*_a_	source
	5.54 × 10^3^	2.81	4600.4	Burgess et al.^43^
	**1.172 × 10**^**1**^	**3.54**	**4739.0**	**P.W. (Ab initio)**
	1.12 × 10^21^	–2.27	2239.6	Burgess et al.^43^
	**3.73 × 10**^**20**^	**-2.27**	**2239.6**	**P.W. (A/3)**
	5.30 × 10^13^	0	0	Burgess et al.^43^
	**2.65 × 10**^**13**^	**0**	**0**	**P.W. (A/2)**

a In italic are the reaction rate coefficients used in model before alterations (originally from Burgess et al.^43^), while the ones in bold correspond to the updated rate constants, (*k* = *AT*^*n*^ exp(−*E*_a_/*RT*)), units in cal, mol, K, and s. P.W. shows the rate constant coefficients.

For the CO oxidation profiles, the model was predicting the trend at the early time of the experiments, corresponding to a time-period where pyrolysis chemistry dominates, as detailed in the Discussion. The reaction in reaction R3 was found to improve the oxidation profiles but did not negatively impact the pyrolysis profiles. The *A* coefficient of reaction R3 was divided by a factor of 3, within the method uncertainty. Although the molecule CF_3_CH_2_ comes directly from pyrolysis, it also splits into CF_2_CO, CF_3_, and CH_2_CF_2_, which are all key molecules in producing CO near the peak during oxidation. This breakdown is analyzed further in the Discussion; however, to summarize, if the *A* coefficient in reaction R3 was reduced, there would be a larger amount of the reactant molecules available for other pathways producing more CO.
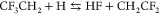
R3

The final alteration made to the model was to improve upon the peak OH* delay times, having calculated many new reaction rates that have a further impact on OH* results. To bring the model more in line with what was seen experimentally, a sensitivity analysis was carried out. The most impactful change to the model was to divide the *A* coefficient of the reaction shown below as reaction R4 by 2.
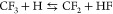
R4

This alteration was made within uncertainty limits; however, it must be noted that further alterations continue to change the model predictions for both the better in some cases and worse in others. This behavior is discussed and analyzed further in the Discussion. The improvements presented above are summarized in Table 5.

## 3 Results and Discussion

### 3.1 CO Time-History Profiles

**Figure 6:**
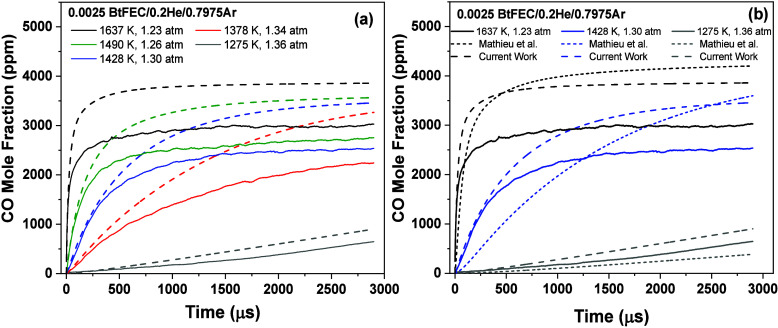
CO time-history profiles for BtFEC pyrolysis from ref (18). (a) Current model (dashed lines in same color as experiment) for temperature range 1275–1637 K compared with experimentally obtained profiles (solid lines). (b) Comparison of previous model from Mathieu et al. (dotted lines)^18^ with new model from current work for temperatures of 1275, 1428, and 1637 K against experimentally obtained time histories.

The comparisons between the experimental CO profiles for the pyrolysis of BtFEC and the predictions from the present model are visible in Figure 6. Figure 6a shows that the current model predicts the initial increase in the CO concentration with high accuracy for intermediate to high temperatures. All profiles have an initial increase followed by a plateau in the levels of CO for temperatures above 1380 K in our conditions and with the test time of the shock tube. The model is over-reactive for all temperatures below ∼1450 K and therefore overpredictive for CO concentrations. This discrepancy is most prominent at 1378 K, where at 1200 μs, the model predicts 40% more CO than what was found experimentally. The plateau predicted by the model is higher than experimental profiles in all cases, and the model was found to be overpredicting the CO concentration at the plateau by approximately 27% at the highest temperature investigated. However, as shown in Figure 6b, this predicted plateau is improved for intermediate and high temperatures compared to the previous model available in the literature.^18^ The overall shape of the current model is also comparable to experimental profiles for all temperatures investigated, an improvement on the previous model, where a steadier increase was predicted at intermediate temperatures. This shape improvement is seen more in intermediate and high temperatures, where the sharp initial increase of CO is well-captured by the current model, in contrast with the much slower increase shown by the previous model.

**Figure 7:**
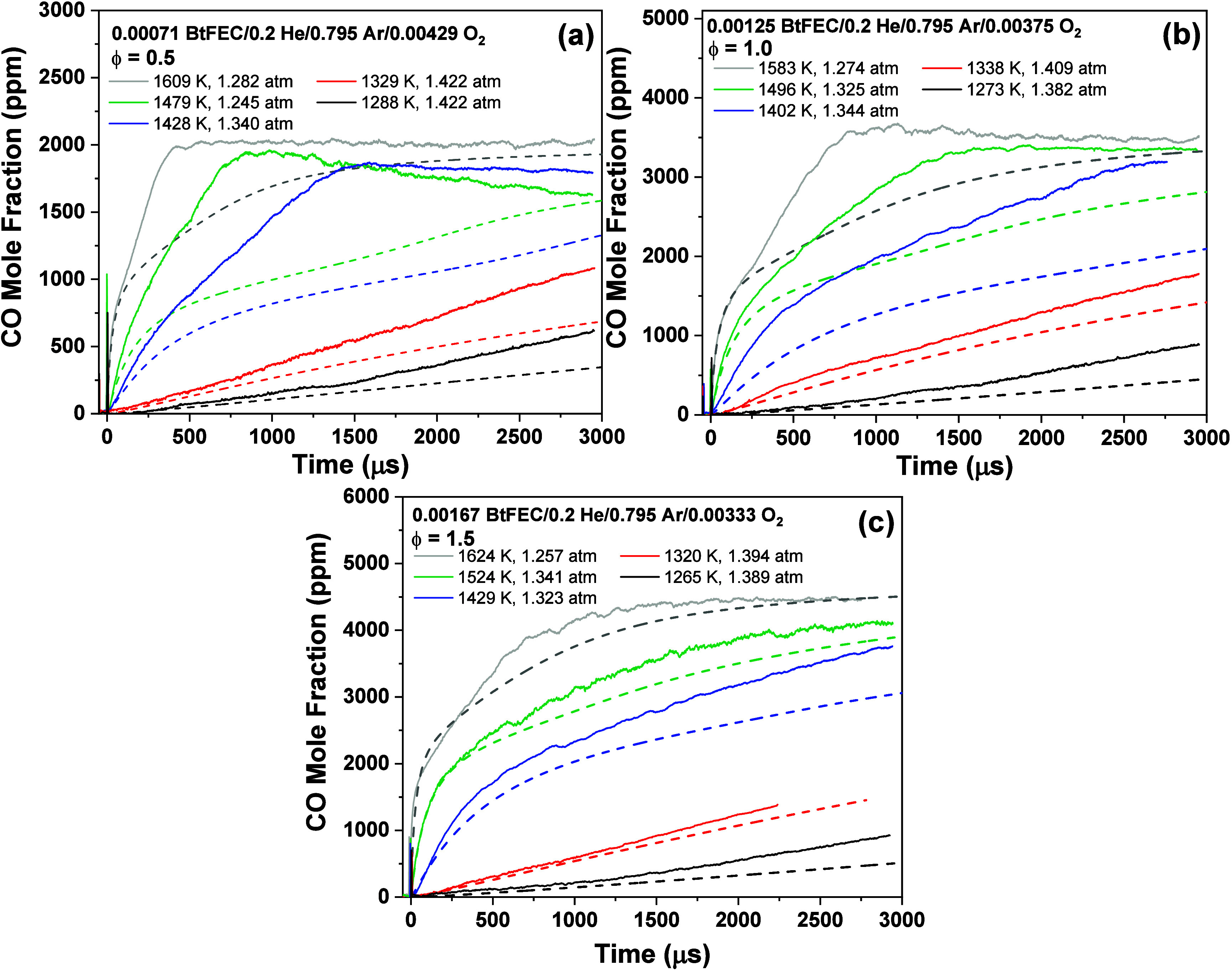
CO profiles for oxidation of BtFEC for a temperature range of 1265–1624 K. Dashed lines represent a model predicting experimental profile of the same color. Three equivalence ratios are shown: (a) ϕ = 0.5, (b) ϕ = 1.0, and (c) ϕ = 1.5. Mixture compositions are indicated in each case.

Figure 7 shows that for oxidation, the model underpredicts the CO level for all temperatures and equivalence ratios within the time frame investigated. However, according to the model, the shape of the profiles shows an initial and rapid formation of CO via BtFEC pyrolysis, followed by a higher poorly defined peak or even plateau due to oxidation. The model mostly predicts the initial pyrolysis portion with high accuracy due to the improvements discussed above. At lower temperatures, the model more accurately predicts the CO profiles, only deviating approximately halfway through the test time to slightly underpredict CO concentrations. This later-time behavior is due to the oxidation occurring later in the test time, as lower temperatures cause a less reactive system. For the fuel-lean case, i.e., ϕ = 0.5 shown in Figure 6a, a rapid increase is observed, closely followed by a plateau. This plateau is not typically encountered for fuel-lean conditions for CO time histories from simple hydrocarbon fuel oxidation, where the CO profile typically presents a peak associated with the CO-to-CO_2_ conversion, seeing as there is excess amounts of oxygen available in the mixture. The chemistry behind this plateau is investigated further in the Discussion.

The model at equivalence ratio ϕ = 0.5 is highly underpredictive for high and intermediate temperatures by a factor of nearly 0.5 at the point of inflection between the sharp increase in CO concentration and the plateau for the highest temperature. The plateau however is better captured by the model in the case of the highest temperature with only a 5% difference in model compared to experiment. For the stoichiometric condition, Figure 7b, the two highest temperatures also show the plateau discussed for the fuel-lean case. This plateau is not seen for lower temperatures due to the test time limitation. For the highest temperature, the model similarly underpredicts the high point of inflection before the plateau by a factor of 0.3. Although this feature is significantly lower on the corresponding profile predicted by the model, the plateau reached is underestimated by approximately 8%, indicating that the end CO concentration is accurately captured by the model.

In the case of the fuel-rich mixture, Figure 7c, the model is aligned more with what was seen experimentally; a testament of the improvements made to the pyrolysis chemistry. At the highest temperature, the inflection point discussed for the lean and stoichiometric cases from the model slightly underpredicts by a factor of 0.1, indicating a much closer prediction. The end CO concentrations at lower temperatures are captured well by the model, and at the highest temperature, the model and experiment give the same plateau. The lower temperatures also show a much better agreement with the model, as these conditions are mainly governed by BtFEC pyrolysis. Overall, CO profiles are well represented initially by the model as well as toward the end of the test time. The oxidation peak is largely underestimated by the model, which is discussed in further detail in the Discussion.

### 3.2 OH* Peak Times

τ_OH*_ measurements were carried out for three equivalence ratios for three different types of mixtures. The first type of mixture corresponds to the mixtures used for the CO time-history measurements during oxidation, as described above, where the OH* profiles were taken simultaneously with the CO measurements. For these BtFEC/O_2_/He/Ar mixtures, the three equivalence ratios were ϕ = 1.5, 1.0, and 0.5. The second and third types of mixtures were H_2_/O_2_/Ar and CH_4_/O_2_/Ar mixtures seeded with BtFEC (10% of initial fuel concentration) over a full range of equivalence ratios.^18^ For these seeded mixtures, the τ_OH*_ measurements were obtained for the equivalence ratios of ϕ = 0.5, 1.0, and 2.0. Figure 7 shows the τ_OH*_ predicted by the model from the present work for different mixtures, compared with the previously published model.^18^ For the BtFEC/O_2_ mixtures shown in Figure 7a, the model’s predictions are comparable with those from the previously published model at low and high temperatures but shows a slightly increased τ_OH*_ for intermediate temperatures. The model shows the classic “high temperature” trend of decreasing τ_OH*_ with increasing temperature but does not capture the strong equivalence ratio effect seen experimentally. As a result, while the model is somewhat close to the data for the fuel-rich case (overestimating high and intermediate temperature times by a factor of only 1.2), it does not capture the reduced τ_OH*_ with lower equivalence ratios. For instance, for equivalence ratios of ϕ = 1.0 and ϕ = 0.5, larger differences by factors of 1.6 and 2.7 were observed compared to the experimental data points, respectively. Figure 7b shows the difference between the model and experimental τ_OH*_ for BtFEC oxidation in Ar/H_2_ mixtures for the three equivalence ratios investigated. Again, the model captures the trend that at higher temperatures the τ_OH*_ decreases but does not capture the increase in τ_OH*_ with the equivalence ratio.

**Figure 8:**
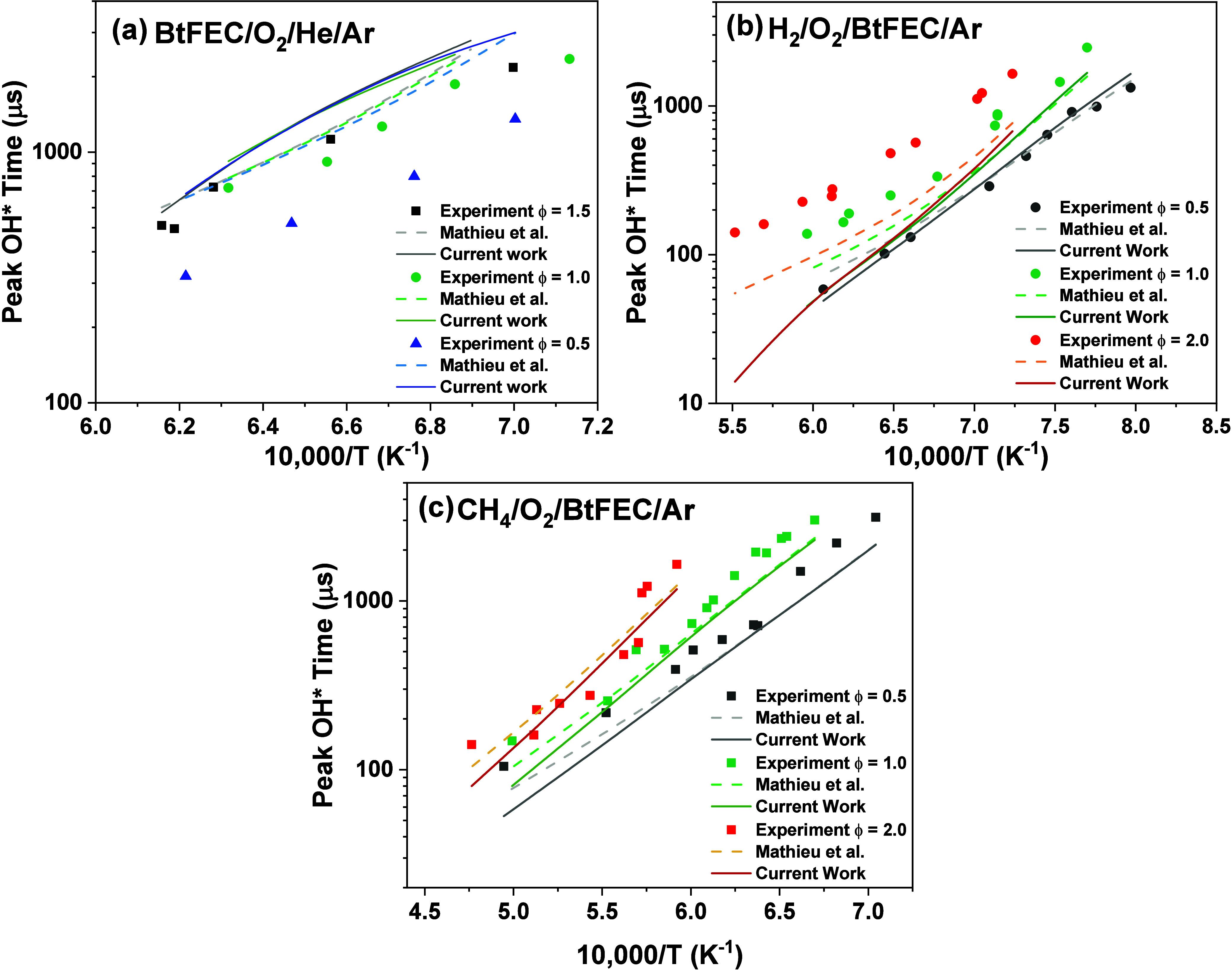
Peak OH* time for current work and previous model from Mathieu et al.^18^ compared to experimental data. (a) Mixtures of BtFEC/O_2_/He/Ar for three equivalence ratios (ϕ = 0.5, 1.0, and 1.5). (b) Mixtures of H_2_/O_2_/BtFEC/Ar for three equivalence ratios (ϕ = 0.5, 1.0, and 2.0). (c) Mixtures of CH_4_/O_2_/BtFEC/Ar for three equivalence ratios (ϕ = 0.5, 1.0, and 2.0).

The model at ϕ = 0.5 predicts the experimental data well (within a factor of 1.3), with similar predictions to those of the original model but aligning with the slightly higher predictions for the lower temperatures investigated. For ϕ = 1.0, the current model shows a similar prediction to the previous model for the majority of the temperature range but predicts a lower peak delay time for higher temperatures which is not seen experimentally. However, this moderately accurate prediction of the model is not the case for the ϕ = 2.0 condition. This fuel-rich behavior is an interesting result, as the fuel-rich case for the BtFEC/O_2_ mixture was predicted well by the model for CO time histories. This trend indicates that the discrepancy pertains to more of the base chemistry of H/O/F in the model, which is in need of updating for more accurate predictions. Figure 7c shows the three equivalence ratios for the BtFEC/CH_4_ mixtures. The model predicts the trends found experimentally relatively well with similar accuracy to Mathieu et al.^18^ overall. For all conditions, a higher peak delay time is predicted by the current model compared with the literature, which is more in line with experimental data for all three conditions (Figure 8).

### 3.3 Speciation in MFR Using FTIR

**Figure 9:**
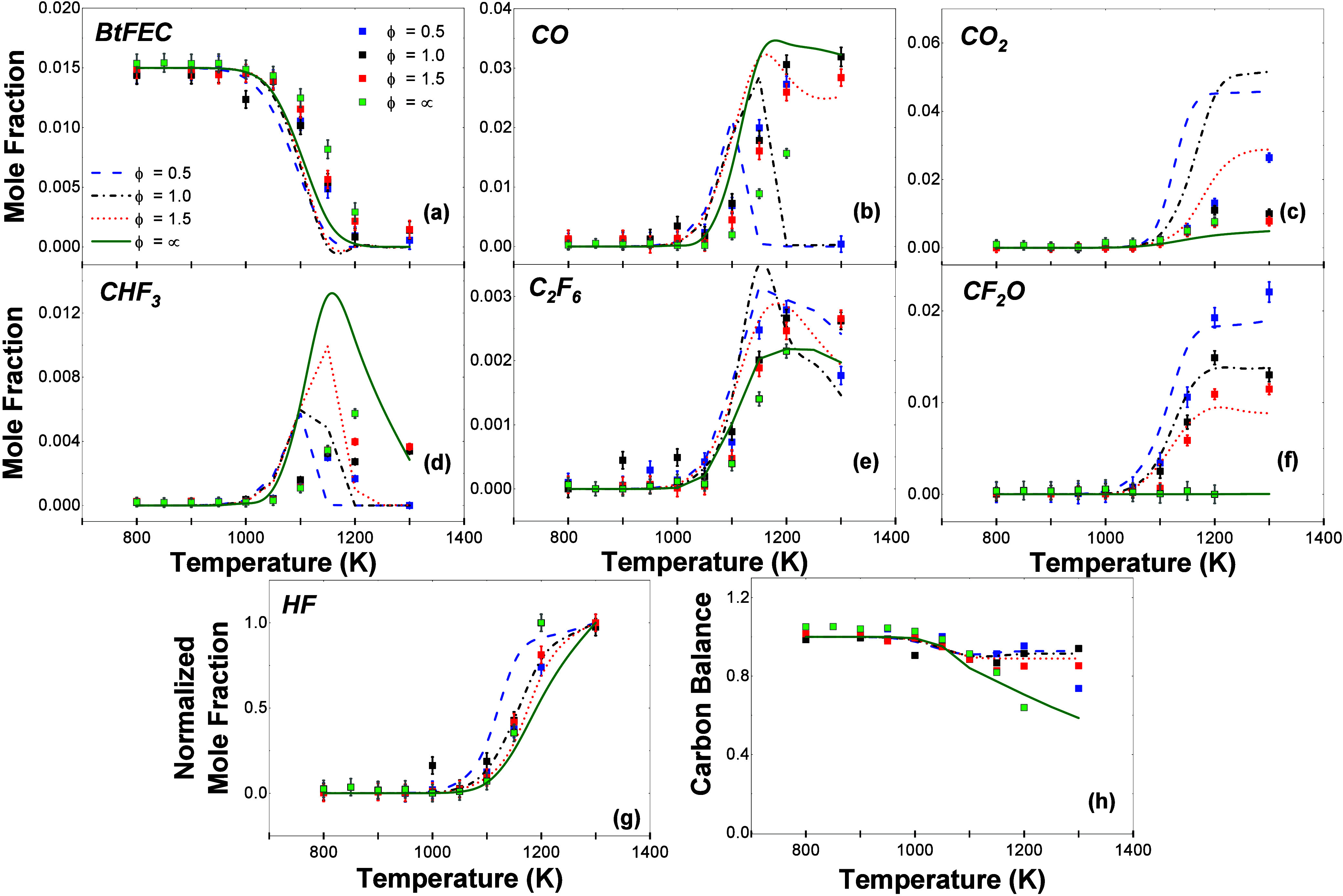
Measured mole fractions of (a) BtFEC, (b) CO, (c) CO_2_, (d) CHF_3_, (e) C_2_F_6_, (f) CF_2_O, and (g) normalized HF for BtFEC pyrolysis (ϕ = ∞) and oxidation (ϕ = 0.5, 1.0, and 1.5) conditions in the temperature range 800–1300 K. (h) Carbon balance. Lines of differing dashes represent the computed mole fractions from the current mechanism for each equivalence ratio.

Speciation results from the MFR measurements are shown in Figure 9 for seven different species (BtFEC, CO, CO_2_, CHF_3_, C_2_F_6_, CF_3_O, and normalized HF) as well as a carbon balance. In oxidation conditions, the trend predicted by the model matches the measurements for BtFEC, whereby there is no decrease in BtFEC mole fraction at lower temperatures, a steady decrease for the temperature range 1000–1200 K until the BtFEC and related species have been fully oxidized in the reactor. This trend is the case for all equivalence ratios; however, the decrease in the BtFEC mole fraction at each temperature varies with the equivalence ratio. The model also shows that BtFEC starts oxidizing at 1000 K and that the molecule is fully consumed at 1150 K for all equivalence ratios. This is a lower temperature than shown by the experimental data as BtFEC starts to oxidize at 1050 K and is fully consumed by 1300 K for ϕ = 0.5 and not yet fully consumed for ϕ = 1.0 and 1.5. However, under pyrolysis conditions, the molecule starts to break up at 1000 K, in line with the experimental data, but is fully consumed by 1200 K, where experimentally there was still an appreciable amount of BtFEC.

The model overall predicts the trends of all other speciation measurements across the temperature range 800–1300 K relatively well with discrepancies mainly for CO, CO_2_, and CHF_3_. For the CO profiles, there is an increase in the amount of CO at higher temperatures starting from 950 K. For ϕ = 0.5, a peak is reached at 1200 K, and the concentration returns to a mole fraction of zero by 1300 K. This peak is captured by the model; however, it is reached at a lower temperature of 1100 K with a similar peak mole fraction and the concentration returns to a mole fraction of 0 by 1150 K. The highest measured mole fractions were for high temperatures at equivalence ratio ϕ = 1.0. This magnitude of mole fraction is captured by the model, but the peak is again reached at a lower temperature. For equivalence ratio ϕ = 1.5, the highest measured CO mole fraction is lower than ϕ = 1.0 for the temperature range investigated here; however, from the trend of an increasing equivalence ratio giving higher mole fraction peaks, the peak for fuel rich conditions can be assumed to be reached at higher temperatures.

The CO_2_ profiles given by the model are highly overpredictive for all oxidation conditions; however, they match pyrolysis measurements relatively well. Experimental results indicate that, under fuel-lean conditions, there is the highest concentration of CO_2_ present at temperatures of 1100 K and higher. This high concentration is captured less well by the model, where for stoichiometric conditions the peak is higher than for ϕ = 0.5.

Modeling for CHF_3_ differs from the measurements by both the mole fractions reached and the peak being observed at a lower temperature than what was found experimentally. The order in which the peaks are predicted by the model, *i.e*., ϕ = 0.5 reaching a peak at a lower temperature than stoichiometric and fuel rich conditions is correctly determined with pyrolysis reaching the peak CHF_3_ concentration at the highest temperature. However, the peak is predicted to occur at approximately 100 K lower in temperature than what was found experimentally.

In contrast to the discrepancies of CO, CO_2_, and CHF_3_ between measurements and computations, the model predicts the profiles of C_2_F_6_ and CF_2_O mole fractions and the carbon balance. It must be noted that although there is an expectation for the model of the carbon balance to remain at 100% at different temperatures, there is a small decrease shown both by the experiments and by the model after 1000 K. This decrease is due to the fact the carbon balance for the model has been determined using the species that were detected during the experiments.

### 3.4 Laminar Flame Speed

**Figure 10:**
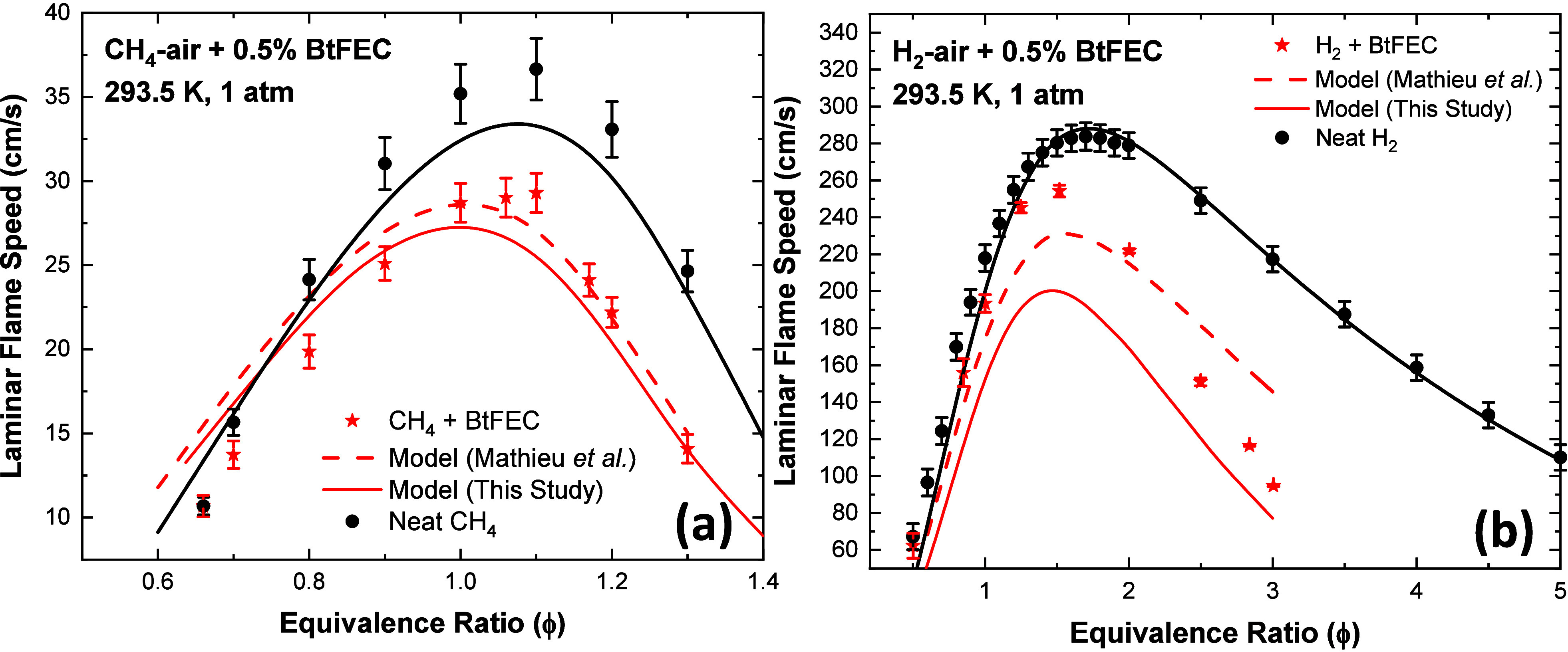
Results of the current model (solid line) in comparison to experimental data for (a) CH_4_ in air seeded with 0.5% BtFEC and neat CH_4_/air from Mathieu et al.^18^ and Turner et al.,^44^ respectively, and (b) H_2_ in air seeded with 0.5% BtFEC and neat H_2_/air from Mathieu et al.^18^ and Krejci et al.^45^ The previous model (dashed line) by Mathieu et al.^18^ is also shown for comparison of the seeded mixtures.

The LFS measurements from our former study^18^ were used to further validate the model as well as provide evidence for the fire suppressant effect of BtFEC. The current model was also compared to the previously published model by Mathieu et al.^18^ The updated model performs well for the CH_4_ mixture shown in Figure 10a, giving overall a slightly lower flame speed than experimentally obtained in stoichiometric and rich conditions, a feature also observed for the neat CH_4_ mixture. On the lean side, the flame speed predicted by the model is higher than what was measured, a behavior also observed in the previously published model. Although the updated model is slightly closer to the measured data on the lean side, it seems to fall short compared to the previous model above an equivalence ratio of ϕ = 0.9. Under stoichiometric conditions, the updated model underestimates the flame speed by 3.5%. The maximum deviation of the model from the data under rich conditions occurs at ϕ = 1.1, where the model underestimates the flame speed by 11.1%. However, this deviation between the model and the data for the BtFEC mixture corresponds to the deviation between the model and the data for the neat mixture (also approximately an 11% underprediction), indicating that the effects of the BtFEC addition are better captured by the revised model. The highest deviation from the model under lean conditions occurs at ϕ = 0.7 where the model overpredicts the flame speed by 25.4%. On the other hand, the model accurately predicts the flame speeds at equivalence ratios of ϕ = 0.9 and ϕ = 1.3 within the experimental uncertainty. The experimental data for the CH_4_ mixture show a decreased flame speed by 18% when 0.5% BtFEC is added to the mixture, mirrored by the model with a predicted decrease of 15%, indicative of the fire-suppressant effect of BtFEC.

The updated model predicts a much lower flame speed to the one presented in Mathieu et al.^18^ for the H_2_-seeded mixture. It shows a largely reduced flame speed compared to what was found experimentally, with the largest deviation at the peak, where ϕ = 1.5, with a 20% under-prediction. The lean-side shows only a slight decrease in the flame speed prediction when compared to the model by Mathieu et al., with again a 20% underprediction compared to the experimental data at ϕ = 1.0. However, the current model shows a much better shape to the curve and an improvement in the predictions for equivalence ratios above 1.5. The current model again indicates a flame speed value 20% under the experimental value at ϕ = 2.8; however, the previous literature model shows an overprediction by 30% after accurately predicting the experimental data point at ϕ = 2. This behavior indicates that the shape of the current model is more consistent with the trend observed experimentally. The model works well for the neat mixture and slightly underpredicts the flame speed on the lean side. This result is also seen in the seeded mixture, which underestimates the lean side; however, this underestimation increases through the stoichiometric region, and the model continues to underestimate the experimental data through the fuel rich side in contrast to the previous model. This underestimation may be explained through the composition of the burned gas. For example, the model predicts a significant amount of the larger molecules such as CF_3_CH_2_OCOOCHCF_3_ still present in the burned gas (443 ppm at ϕ = 0.6 and 354 ppm at ϕ = 3.0), indicating that reactions producing the stable product species have not completely occurred in our system as per the model. These reactions are typically energetic and would influence the flame temperature and therefore the flame speed; partly explaining the discrepancy with the data. Further analysis of LFS in the discussion can be used to explain the results observed in Figure 10.

### 3.5 Discussion

**Figure 11:**
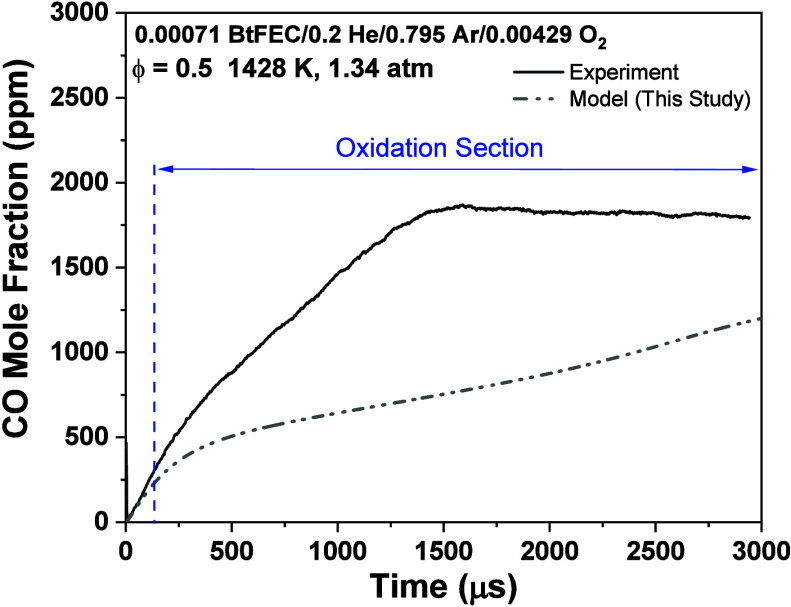
Experimental and modeling CO profiles indicating the oxidation section of the reaction time frame for ϕ = 0.5 at a temperature of 1428 K.

**Figure 12:**
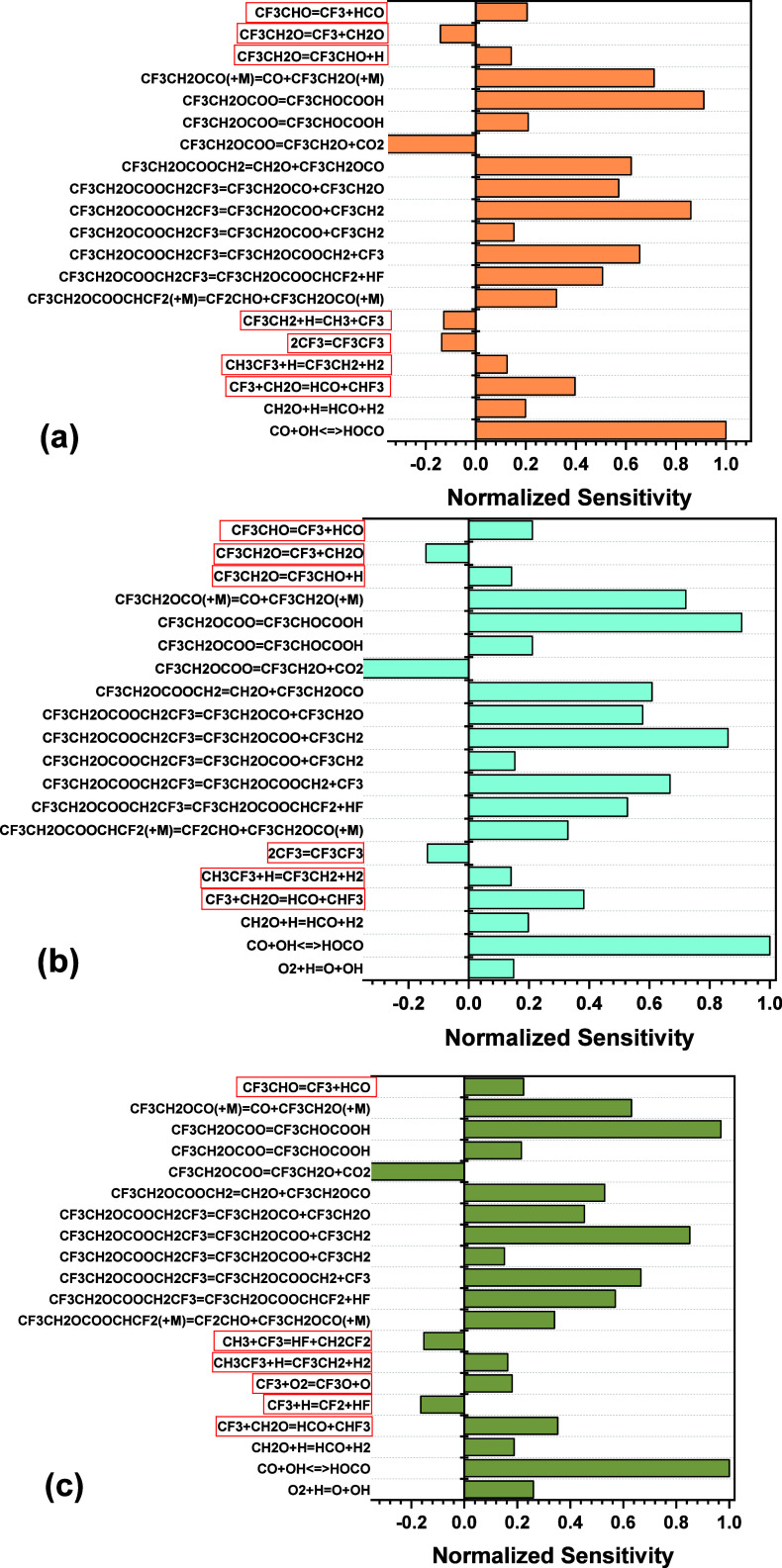
Normalized sensitivity analysis for CO at 1429 K, 1.34 atm at three equivalence ratios (a) ϕ = 1.5, (b) ϕ = 1.0, and (c) ϕ = 0.5. Boxed reactions pertain to those investigated for the oxidation mechanism. Positive coefficients relate to an increase in CO production and negative to a decrease.

CO time histories are very useful to constrain, assess, and validate the chemical kinetic mechanism being used. While the pyrolysis of CO has been accurately captured by the model, the oxidation peak, or lack thereof, for all three equivalence ratios has been underpredicted by the model. In particular, the fuel-lean and stoichiometric conditions are impacted the most, which is also seen in the τ_OH*_ for BtFEC/O_2_ mixtures. To further understand the reasons behind this discrepancy, the oxidation section of the profile must be defined, and a numerical analysis needs to be conducted. An example of this is shown in Figure 11 for an intermediate temperature of 1428 K for the fuel-lean case. A perturbative CO sensitivity analysis using the Ansys Chemkin module was carried out for an intermediate temperature at each equivalence ratio of the mixtures, where CO was measured. To understand all of the reactions contributing to the profile, the top 20 reactions were found for the whole test time. The results of this analysis are shown in Figure 12, where reactions pertaining to the oxidation section are highlighted.

As visible in Figure 12, even in the “oxidation section” of the data, the majority of the sensitive reactions pertain to the pyrolysis mechanism (i.e., where the initial BtFEC molecule breaks into smaller fragments), where the BtFEC pyrolysis reaction rates have been updated for the current model by using *ab initio* calculations. As seen from the results, the pyrolysis section is accurately predicted by the model, indicating that more focus is required for the oxidation section of the CO profile. Oxidation reactions that have already been updated for the current mechanism and that appear in the sensitivity analysis in Figure 12 are shown in reactions R2, R3, and R4 (copied below for clarity). Although the reaction R2 rate coefficients have been newly calculated, reactions R3 and R4 were updated by dividing the *A* coefficient within the uncertainty. The reaction R3*A* coefficient was divided by 3, the furthest deviation possible from the original coefficient given Mathieu et al.^18^ (original source from Burgess et al.^43^) still keeping within uncertainty limits. For reaction R4, the *A* coefficient was divided by 2, which caused a much-improved CO oxidation profile, as well as improved peak delay times. However, any further alteration caused detrimental effects to the CO pyrolysis profile, which is estimated relatively well by the model. This trade-off shows how prominent this reaction and those involving small F-containing molecules such as CF_3_ are in predicting CO mole fractions, underlining the need for more accurate rate constants for the base chemistry involving fluorine reactions.

For all equivalence ratios in Figure 12, there are three reactions, common to all conditions, relating to the oxidation section of the CO profiles. One of these reactions is discussed above (reaction R2), leaving two reactions to investigate, which are outlined in reactions R5 and R6.
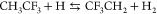
R5
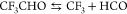
R6

**Figure 13:**
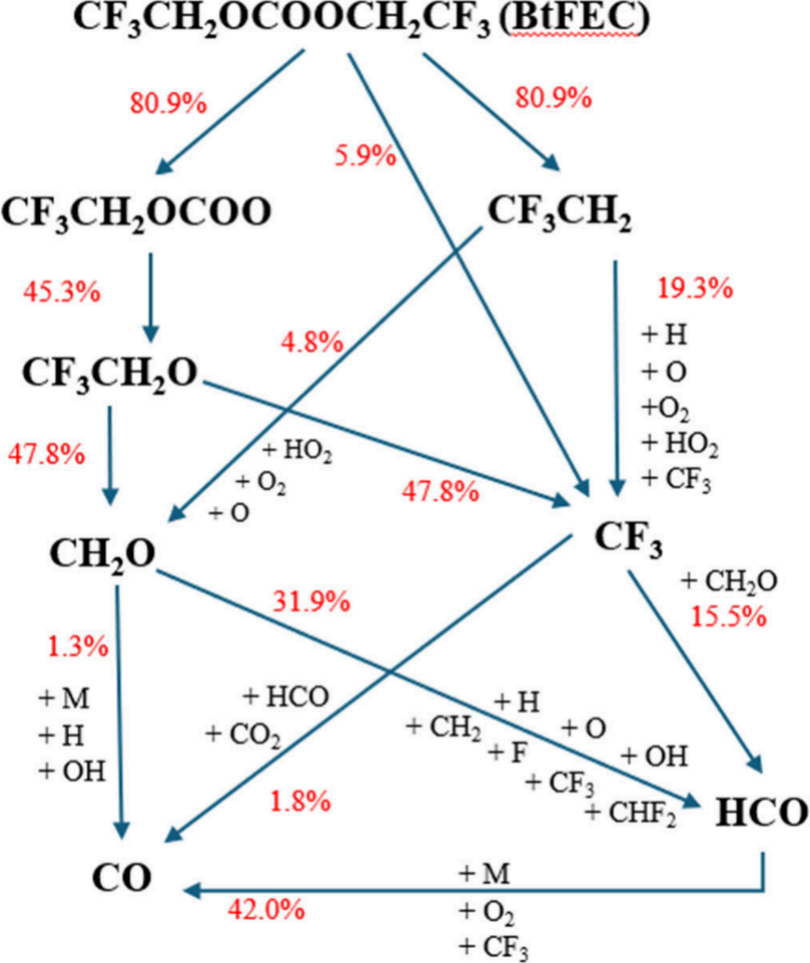
Reaction pathway from BtFEC to CO with relative rate of production percentages for the produced species for ϕ = 0.5, 1428 K, and 1.34 atm. No species added on the arrow indicates a decomposition reaction. The percentage represents the contribution of reactions of the upper species to form the lower species.

The reactions outlined above that were most sensitive to CO production are related to radicals CF_3_CH_2_, CF_3_, and CH_2_CF_2_. Reactions producing CH_2_CF_2_ are seen in the sensitivity analysis to be inhibiting the production of CO as forming CH_2_CF_2_ involves the consumption of CF_3_CH_2_, CF_3_, and CH_3_, which could otherwise be used to form CO (although a small fraction of CH_2_CF_2_ can lead to CO via CH_2_CF_2_ + O ⇄ CHF_2_ + HCO). The radical CF_3_CH_2_O is also heavily involved in CO production by splitting into CH_2_O and CF_3_. The main pathway to CO is by these two species reacting to give HCO, a well-known precursor to CO. To better understand how these molecules are important in the CO production, a reaction pathway analysis was carried out. These molecules were traced from the initial BtFEC molecule through to CO as shown in Figure 13, at 200 μs, just after the oxidation section started, to ensure the oxidation reactions forming CO were involved.

The importance of radicals CF_3_CH_2_ and CF_3_ in CO production can be used to provide potential improvements to the model. Alterations of the rate coefficients for reactions where these molecules are produced (reactions R5 and R6) were conducted through multiplying the *A* coefficient by a factor of 2 (within uncertainty limits). Although these two reactions are sensitive at all equivalence ratios, neither cause a change in either the CO oxidation or pyrolysis profiles. This insensitivity shows that the time history of CO is based on the rates of multiple intertwining reactions instead of purely forming the effective “precursor” molecules. Forming these molecules is only half of the story, and an increase in the rate of subsequent reactions or reactions producing the species needed in these subsequent reactions may cause more of an improvement. This result shows the importance of accurate reaction rates for the base chemistry of fluorinated species as the reactions are highly interconnected.

Note that the chemistry leading to CO is significantly simpler under pyrolysis conditions. According to the model, under the conditions investigated, the chemistry leading to CO formation from BtFEC pyrolysis follows this main pathway:
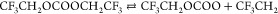
R7
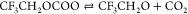
R8
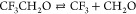
R9
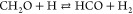
R10
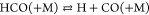
R11

Note that additional minor pathways are also predicted:
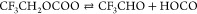
R12
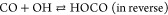
R13
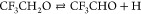
R14

A similar analysis was performed at a lower temperature to illustrate the difference in the reaction pathway for BtFEC consumption. The figure is available in the Supporting Information and, basically, shows that for the same consumption level for BtFEC as in Figure 13, BtFEC is chiefly consumed via H-radical abstraction. The resulting radical, CH_3_CH_2_OCOOCHCF_3_, then decomposes into CF_3_CH_2_OCO and CF_3_CHO. The CF_3_CH_2_OCO then decomposes into CO and CF_3_CH_2_O; the latter will then form CO following the same pathway as identified in Figure 13. The other fragment, CF_3_CHO, will react with a radical to form CF_3_CO, which will thermally decompose to directly form CO.

**Figure 14:**
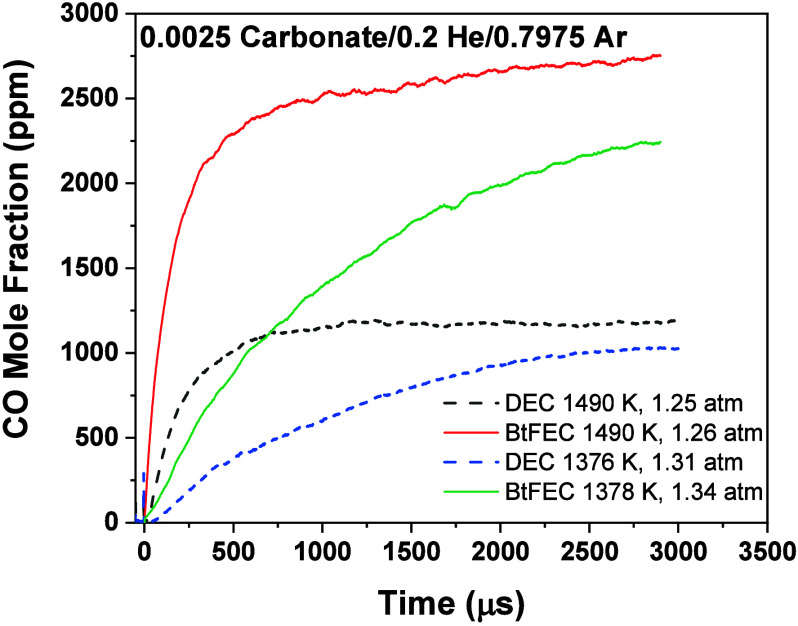
Comparison of the CO profiles for BtFEC and DEC^46^ at similar temperatures and pressures for pyrolysis conditions. Dashed lines are used to show the profiles obtained from the pyrolysis of the DEC.

The CO profiles obtained for BtFEC during this study (oxidation) and in Mathieu et al.^18^ (pyrolysis) can be compared with those obtained for DEC in Grégoire et al.^46^ (pyrolysis) and Cooper et al.^46^ (oxidation). Comparing CO profiles for DEC and BtFEC for similar conditions in pyrolysis, Figure 14, shows that BtFEC forms a significantly higher mole fraction of CO compared to DEC from the same initial mole fraction of fuel. The details of the CO formation chemistries are provided in their respective papers but, in essence, the fact that BtFEC produces more CO than DEC is due to a combination of factors linked to the presence of F atoms in the BtFEC structure. First, these molecular F atoms tend to form HF as a stable product. With more F than H atoms in BtFEC, this leads to a higher availability of C and O atoms to form CO than DEC, where various hydrocarbons and H_2_O are also formed. Second, the pyrolysis decomposition of DEC is controlled by molecular elimination reactions, resulting in only ethanol as a precursor of CO. On the other hand, BtFEC pyrolysis is driven by decomposition channels (Figure 13) with CF_3_CH_2_O and CF_3_CHO as the main CO precursors. Since the C–F bound dissociation energy is larger than the C–H one (by about 5 kcal.mol^–1^), the chemistry of these precursors is thus oriented toward the oxygenated section of the molecules in contrast to DEC where reactions with the terminal carbon atoms (CH_3_ extremities) can take place.

**Figure 15:**
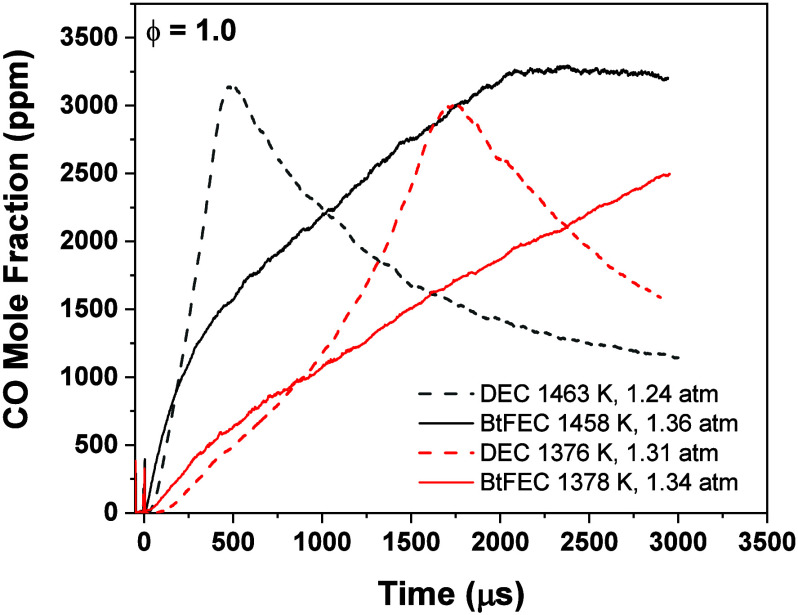
CO profiles for BtFEC compared with those obtained for DEC^46^ at similar temperatures and near atmospheric pressures in stoichiometric conditions. Dashed lines represent the profiles from DEC, and solid lines represent BtFEC.

For the comparison at stoichiometric conditions, Figure 15, the level of CO cannot be directly compared since the dilution level is different between the two studies (99.5% for BtFEC against 99.25% for the DEC study from Cooper et al.^46^) and since the species considered as final combustion products are different, leading to different fuel and oxygen concentrations. However, the shape of the profiles is very different, with a near plateau after the initial CO formation period for BtFEC and a peak in the CO signal for DEC. This peak for DEC is due to the oxidation of CO into CO_2_ primarily via CO + OH ⇄ CO_2_ + H. For the BtFEC case, a numerical analysis shows that this oxidation process of CO by OH is also happening, but at a much lower scale: according to the model, the amount of OH radicals formed with BtFEC is more than 100 times lower than during DEC oxidation for similar conditions (ϕ = 1.0, 99.5% dilution, 1450 K, 1.3 atm). Again, the F atoms in molecules and radicals tend to take the small amount of molecular H atoms in BtFEC away to form HF, which then limits the amount of OH radicals.

**Figure 16:**
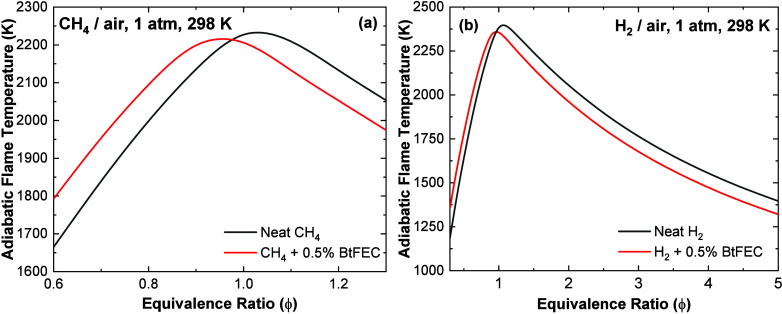
Adiabatic flame temperature for neat mixtures compared with those of BtFEC-seeded mixtures for a range of equivalence ratios.

Last, further understanding of the underlying chemistry can be found through the analysis of the LFS. Importantly, the effects of BtFEC on the LFS seem to be due to two contributing aspects: the flame temperature and the flame-inhibiting chemistry. The flame-inhibiting chemistry is essentially due to the trapping of H radicals by the F atom, forming the stable HF molecule and preventing the important branching reaction H + O_2_ ⇄ O + OH to occur. To visualize the flame temperature effect, an adiabatic flame temperature calculation was carried out for both the neat and seeded mixtures using the current model, and the results are shown in Figure 16. The addition of BtFEC decreases the peak adiabatic flame temperature, which will also tend to decrease the LFS, but it is important to mention that the adiabatic flame temperature profile is shifted toward the lean side. Under rich conditions, the adiabatic flame temperature is then reduced by the addition of the BtFEC causing an overall decrease of the LFS in addition to the chemical mechanism described above.

However, on the lean side, the adiabatic flame temperature is significantly higher when BtFEC is added, by more than 100 K below ϕ = 0.85 for both seeded mixtures, which counterbalances the chemical effect, causing only a small reduction in the LFS observed experimentally, which was not fully captured by the model. The lower flame speed on the rich side of the H_2_-seeded mixture captured by the model, and experiments can also be attributed to this lower adiabatic flame speed. Note that these results concern the data and conditions from the present study, where only 0.5% BtFEC was added to the baseline mixture. It is expected that, compared to the baseline, a decrease in the laminar flame speed will be observed over the entire range of equivalence ratios investigated at larger BtFEC concentrations. This is seen in Grégoire et al.^47^ where higher concentrations of BtFEC (up to 1.4%) in a mixture with ethyl methyl carbonate reduced the laminar flame speed by approximately 40%.

## 4 Conclusions

An improved mechanism for the candidate LIB fire suppressant BtFEC was presented, and its performance was evaluated against a variety of experimental measurements. Shock-tube experiments were carried out to obtain CO time-history profiles for both pyrolysis and oxidation of BtFEC with equivalence ratios ϕ = 0.5, 1.0, and 1.5 and a temperature range of ∼1200–1650 K at near-atmospheric pressures. τ_OH*_ peak delay times were also obtained through this method by capturing OH* chemiluminescence, and both sets of data were compared with the current model as well as the previously published mechanism by Mathieu et al.^18^ The current mechanism was more aligned with experimental data for mid to high temperatures in CO profiles and performed overall similarly to the previous model for τ_OH*_ with the exception of the rich seeded H_2_ mixture, where the model predicted shorter peak delay times.

Species measurements were carried out with the use of an MFR with a controlled temperature profile, and the results were compared with the current mechanism for the first time. The model captured the trends of concentration against temperature for the majority of species relatively well; however, the concentrations of CO_2_ and CHF_3_ predicted were too high compared to the data. LFS measurements from Mathieu et al.^18^ were used to compare the current mechanism with the initial model and showed that, considering deviations from the base mechanism, the chemistry of the new model represents the data well for the CH_4_ mixture. The shape of the LFS curve for the H_2_ mixture is improved compared to the initial model by Mathieu et al.;^18^ however the values are consistently 20% below what were seen experimentally.

Analysis of sensitive reactions for the CO profiles allowed for key reactions to be investigated further to identify possible improvements to the model. Key features of the CO profiles were discussed in relation to the underlying chemistry. Deviations in the LFS were considered by comparing the seeded mixture with the neat CH_4_ and H_2_ mixtures. Underprediction in the model for near-stoichiometric and rich conditions was determined to be the result of the base mechanism for CH_4_ mixtures. Overprediction on the lean side for the seeded mixture was found to be the result of a higher adiabatic flame temperature given by the model, therefore dominating the underlying chemistry. H_2_ mixtures were well represented by the model terms of the trend with a consistent underprediction in flame speed. This level of agreement can be attributed to more of the fundamental H/F chemistry that has been pointed out that needs updating.

## Supporting Information Available

The model’s updated and added reactions and their rate coefficients, figures presenting all the CO data, as well as tables for the ignition and induction delay time and MFR data (PDF)
Data for the CO profiles under oxidation conditions (XLSX)
Files for the detailed kinetics model (kinetic mechanism) (TXT)
Files for the detailed kinetics model (thermodynamic data) (TXT)
Files for the detailed kinetics model (transport data) (TXT)

## References

[ref1] Wang Q.; Mao B.; Stoliarov S. I.; Sun J. A review of lithium ion battery failure mechanisms and fire prevention strategies. Prog. Energy Combust. Sci. 2019, 73, 95–131. 10.1016/j.pecs.2019.03.002.

[ref2] Liu Z.; Liu Z.; Li K.; Zhao X.; Chen M.; Miao H.; Xia L. Exploring Trimethyl-Phosphate-Based Electrolytes without a Carbonyl Group for Li-Rich Layered Oxide Positive Electrodes in Lithium-Ion Batteries. J. Phys. Chem. Lett. 2022, 13 (48), 11307–11316. 10.1021/acs.jpclett.2c02585. 36453838

[ref3] Wang X.; Yasukawa E.; Kasuya S. Nonflammable Trimethyl Phosphate Solvent-Containing Electrolytes for Lithium-Ion Batteries: I. Fundamental Properties. J. Electrochem. Soc. 2001, 148 (10), A1058 10.1149/1.1397773.

[ref4] Jia H.; Xu Y.; Zhang X.; Burton S. D.; Gao P.; Matthews B. E.; Engelhard M. H.; Han K. S.; Zhong L.; Wang C.; Xu W. Advanced Low-Flammable Electrolytes for Stable Operation of High-Voltage Lithium-Ion Batteries. Angew. Chem., Int. Ed. Engl. 2021, 60 (23), 12999–13006. 10.1002/anie.202102403. 33783105

[ref5] Li Z. D.; Zhang Y. C.; Xiang H. F.; Ma X. H.; Yuan Q. F.; Wang Q. S.; Chen C. H. Trimethyl phosphite as an electrolyte additive for high-voltage lithium-ion batteries using lithium-rich layered oxide cathode. J. Power Sources 2013, 240, 471–475. 10.1016/j.jpowsour.2013.04.038.

[ref6] Jiang L.; Liang C.; Li H.; Wang Q.; Sun J. Safer Triethyl-Phosphate-Based Electrolyte Enables Nonflammable and High-Temperature Endurance for a Lithium Ion Battery. ACS Appl. Energy Mater. 2020, 3 (2), 1719–1729. 10.1021/acsaem.9b02188.

[ref7] Shim E.-G.; Nam T.-H.; Kim J.-G.; Kim H.-S.; Moon S.-I. Electrochemical performance of lithium-ion batteries with triphenylphosphate as a flame-retardant additive. J. Power Sources 2007, 172 (2), 919–924. 10.1016/j.jpowsour.2007.04.088.

[ref8] Jung K.; Oh S. H.; Yim T. Triphenyl phosphate as an Efficient Electrolyte Additive for Ni-rich NCM Cathode Materials. J. Electrochem. Sci. Technol. 2021, 12 (1), 67–73. 10.33961/jecst.2020.00850.

[ref9] Sikes T.; Mathieu O.; Kulatilaka W. D.; Mannan M. S.; Petersen E. L. Laminar flame speeds of DEMP, DMMP, and TEP added to H2- and CH4-air mixtures. Proc. Combust. Inst. 2019, 37 (3), 3775–3781. 10.1016/j.proci.2018.05.042.

[ref10] Mathieu O.; Kulatilaka W. D.; Petersen E. L. Experimental and modeling study on the effects of dimethyl methylphosphonate (DMMP) addition on H2, CH4, and C2H4 ignition. Combust. Flame 2018, 191, 320–334. 10.1016/j.combustflame.2018.01.020.

[ref11] Li S.; Luo Z.; Li L.; Hu J.; Zou G.; Hou H.; Ji X. Recent progress on electrolyte additives for stable lithium metal anode. Energy Storage Mater. 2020, 32, 306–319. 10.1016/j.ensm.2020.07.008.

[ref12] Zhang X.-Q.; Cheng X.-B.; Chen X.; Yan C.; Zhang Q. Fluoroethylene Carbonate Additives to Render Uniform Li Deposits in Lithium Metal Batteries. Adv. Funct. Mater. 2017, 27 (10), 1605989 10.1002/adfm.201605989.

[ref13] Tu H.; Li S.; Liu C.; Luo Z.; Ni L.; Zhang Y.; Deng W.; Zou G.; Zhou L.; Hou H.; Ji X. Difluoroethylene Carbonate as an Electrolyte Additive for Engineering the Electrolyte–Electrode Interphase of Lithium Metal Batteries. ACS Appl. Mater. Interfaces 2023, 15 (46), 53533–53539. 10.1021/acsami.3c13096. 37938031

[ref14] Pham H. Q.; Lee H.-Y.; Hwang E.-H.; Kwon Y.-G.; Song S.-W. Non-flammable organic liquid electrolyte for high-safety and high-energy density Li-ion batteries. J. Power Sources 2018, 404, 13–19. 10.1016/j.jpowsour.2018.09.075.

[ref15] Smart M. C.; Ratnakumar B. V.; Ryan-Mowrey V. S.; Surampudi S.; Prakash G. K. S.; Hu J.; Cheung I. Improved performance of lithium-ion cells with the use of fluorinated carbonate-based electrolytes. J. Power Sources 2003, 119–121, 359–367. 10.1016/S0378-7753(03)00266-0.

[ref16] Zheng X.; Liao Y.; Zhang Z.; Zhu J.; Ren F.; He H.; Xiang Y.; Zheng Y.; Yang Y. Exploring high-voltage fluorinated carbonate electrolytes for LiNi0.5Mn1.5O4 cathode in Li-ion batteries. J. Energy Chem. 2020, 42, 62–70. 10.1016/j.jechem.2019.05.023.

[ref17] Demelash F.; Gomez-Martin A.; Heidrich B.; Adhitama E.; Harte P.; Javed A.; Arifiadi A.; Bela M. M.; Yan P.; Harte P.; Diddens D.; Winter M.; Niehoff P. Fluoroethylene Carbonate: Bis(2,2,2,) Trifluoroethyl Carbonate as High Performance Electrolyte Solvent Blend for High Voltage Application in NMC811|| Silicon Oxide-Graphite Lithium Ion Cells. Small Struct. 2024, 5 (9), 2400063 10.1002/sstr.202400063.

[ref18] Mathieu O.; Dievart P.; Turner M. A.; Mohr D. J.; Grégoire C.; Alturaifi S. A.; Catoire L.; Petersen E. L. Experimental and Detailed Kinetics Modeling Study of the Fire Suppressant Properties of Di(2,2,2trifluoroethyl) Carbonate. Proc. Combust. Inst. 2023, 39 (1), 499–510. 10.1016/j.proci.2022.07.078.

[ref19] Petersen E. L.; Rickard M. J. A.; Crofton M. W.; Abbey E. D.; Traum M. J.; Kalitan D. M. A facility for gas- and condensed-phase measurements behind shock waves. Meas. Sci. Technol. 2005, 16 (9), 1716 10.1088/0957-0233/16/9/003.

[ref20] Lipkowicz J. T.; Nativel D.; Cooper S.; Wlokas I.; Fikri M.; Petersen E.; Schulz C.; Kempf A. M. Numerical Investigation of Remote Ignition in Shock Tubes. Flow, Turbul. Combust. 2021, 106 (2), 471–498. 10.1007/s10494-020-00219-w.

[ref21] Mathieu O.; Mulvihill C. R.; Petersen E. L. Assessment of modern detailed kinetics mechanisms to predict CO formation from methane combustion using shock-tube laser-absorption measurements. Fuel 2019, 236, 1164–1180. 10.1016/j.fuel.2018.09.029.

[ref22] Cooper S. P.; Grégoire C. M.; Mohr D. J.; Mathieu O.; Alturaifi S. A.; Petersen E. L. An Experimental Kinetics Study of Isopropanol Pyrolysis and Oxidation behind Reflected Shock Waves. Energies 2021, 14 (20), 6808 10.3390/en14206808.

[ref23] Spearrin R. M.; Goldenstein C. S.; Jeffries J. B.; Hanson R. K. Quantum cascade laser absorption sensor for carbon monoxide in high-pressure gases using wavelength modulation spectroscopy. Appl. Opt. 2014, 53 (9), 1938–1946. 10.1364/AO.53.001938. 24663473

[ref24] Grégoire C. M.; Mathieu O.; Petersen E. L. High-temperature line strengths with He- and Ar-broadening coefficients of the P(20) line in the 1 < -- 0 band of carbon monoxide. Appl. Phys. B: Laser Opt. 2023, 129 (12), 187 10.1007/s00340-023-08132-6.

[ref25] Grégoire C. M.; Petersen E. L.; Mathieu O. Shock-Tube CO Measurements during the Pyrolysis of Ethylene Carbonate. Combust. Flame 2023, 257 (2), 113019 10.1016/j.combustflame.2023.113019.

[ref26] Maruta K.; Kataoka T.; Kim N.; Minaev S.; Fursenko R. Characteristics of combustion in a narrow channel with a temperature gradient. Proc. Combust. Inst. 2005, 30, 2429–2436. 10.1016/j.proci.2004.08.245.

[ref27] Kanayama K.; Takahashi S.; Morikura S.; Nakamura H.; Tezuka T.; Maruta K. Study on oxidation and pyrolysis of carbonate esters using a micro flow reactor with a controlled temperature profile. Part I: Reactivities of dimethyl carbonate, ethyl methyl carbonate and diethyl carbonate. Combust. Flame 2022, 237, 111810 10.1016/j.combustflame.2021.111810.

[ref28] Takahashi S.; Nakamura H.; Tezuka T.; Hasegawa S.; Maruta K. Multi-stage oxidation of a CH_2_F_2_/air mixture examined by weak flames in a micro flow reactor with a controlled temperature profile. Combust. Flame 2019, 201, 140–147. 10.1016/j.combustflame.2018.12.014.

[ref29] CanteraMFR, tohoku-edyn. https://github.com/tohoku-edyn/CanteraMFR (accessed 10/22/2024).

[ref30] Frisch M. J. Gaussian 09, Revision D.01; Gaussian, Inc., Wallingford, CT, 2013.

[ref31] Neese F. Software update: the ORCA program system, version 4.0. WIREs Computational Molecular Science 2018, 8 (1), e1327 10.1002/wcms.1327.

[ref32] Kesharwani M. K.; Brauer B.; Martin J. M. Frequency and zero-point vibrational energy scale factors for double-hybrid density functionals (and other selected methods): can anharmonic force fields be avoided?. J. Phys. Chem. A 2015, 119 (9), 1701–14. 10.1021/jp508422u. 25296165

[ref33] Klippenstein S. J.; Georgievskii Y.; Harding L. B. Predictive theory for the combination kinetics of two alkyl radicals. Phys. Chem. Chem. Phys. 2006, 8 (10), 1133–1147. 10.1039/b515914h. 16633594

[ref34] Hassinen E.; Riepponen P.; Blomqvist K.; Kalliorinne K.; Evseev A. M.; Koskikallio J. Kinetics of reactions between methoxycarbonyl, methyl, and methoxy radicals formed in flash photolysis of dimethyl oxalate in gas phase. Int. J. Chem. Kinet. 1985, 17 (10), 1125–1134. 10.1002/kin.550171009.

[ref35] Sivaramakrishnan R.; Michael J. V.; Wagner A. F.; Dawes R.; Jasper A. W.; Harding L. B.; Georgievskii Y.; Klippenstein S. J. Roaming radicals in the thermal decomposition of dimethyl ether: Experiment and theory. Combust. Flame 2011, 158 (4), 618–632. 10.1016/j.combustflame.2010.12.017.

[ref36] Saxena S.; Kiefer J. H.; Klippenstein S. J. A shock-tube and theory study of the dissociation of acetone and subsequent recombination of methyl radicals. Proc. Combust. Inst. 2009, 32 (1), 123–130. 10.1016/j.proci.2008.05.032.

[ref37] Harding L. B.; Georgievskii Y.; Klippenstein S. J. Predictive theory for hydrogen atom-hydrocarbon radical association kinetics. J. Phys. Chem. A 2005, 109 (21), 4646–4656. 10.1021/jp0508608. 16833805

[ref38] Barker J. R.; Stanton J. F.; Aieta C.; Ceotto M.; Gabas F.; Kumar T. J. D.; Li C. G. L.; Lohr L. L.; Maranzana A.; Ortiz N. F.; Preses J. M.; Simmie J. M.; Sonk J. A.; Stimac P. J. MultiWell-2021 Software Suite; Barker J. R., Ed.; University of Michigan: Ann Arbor, Michigan, 2021. https://multiwell.engin.umich.edu.

[ref39] AlAbbad M.; Giri B. R.; Szőri M.; Viskolcz B.; Farooq A. A high temperature kinetic study for the thermal unimolecular decomposition of diethyl carbonate. Chem. Phys. Lett. 2017, 684, 390–396. 10.1016/j.cplett.2017.07.020.

[ref40] Burgess D. R. Jr.; Babushok V. I.; Manion J. A. A chemical kinetic mechanism for combustion and flame propagation of CH_2_F_2_/O_2_/N_2_ mixtures. Inter. J. Chem. Kinet. 2022, 54 (3), 154–187. 10.1002/kin.21549.

[ref41] Sharma S.; Abeywardane K.; Goldsmith C. F. Theory-Based Mechanism for Fluoromethane Combustion I: Thermochemistry and Abstraction Reactions. J. Phys. Chem. A 2023, 127 (6), 1499–1511. 10.1021/acs.jpca.2c06623. 36745864

[ref42] Babushok V. I.; Linteris G. T.; Burgess D. R. Jr; Baker P. T. Hydrocarbon flame inhibition by C_3_H_2_F_3_Br (2-BTP). Combust. Flame 2015, 162 (4), 1104–1112. 10.1016/j.combustflame.2014.10.002.

[ref43] Burgess D. R.; Zachariah M. R.; Tsang W.; Westmoreland P. R. Thermochemical and chemical kinetic data for fluorinated hydrocarbons. Prog. Energy Combust. Sci. 1995, 21 (6), 453–529. 10.1016/0360-1285(95)00009-7.

[ref44] Turner M. A.; Paschal T. T.; Parajuli P.; Kulatilaka W. D.; Petersen E. L. Application of high-speed, species-specific chemiluminescence imaging for laminar flame speed and Markstein length measurements in spherically expanding flames. Exp. Therm. Fluid. Sci. 2021, 129, 110477 10.1016/j.expthermflusci.2021.110477.

[ref45] Krejci M. C.; Mathieu O.; Vissotski A. J.; Ravi S.; Sikes T. G.; Petersen E. L.; Kérmonès A.; Metcalfe W.; Curran H. J. Laminar Flame Speed and Ignition Delay Time Data for the Kinetic Modeling of Hydrogen and Syngas Fuel Blends. J. Eng. Gas Turbine Power 2013, 135 (2), 021503 10.1115/1.4007737.

[ref46] Grégoire C. M.; Cooper S. P.; Khan-Ghauri M.; Alturaifi S. A.; Petersen E. L.; Mathieu O. Pyrolysis study of dimethyl carbonate, diethyl carbonate, and ethyl methyl carbonate using shock-tube spectroscopic CO measurements and chemical kinetics investigation. Combust. Flame 2023, 249, 112594 10.1016/j.combustflame.2022.112594.

[ref47] Grégoire C. M.; Almarzooq Y. M.; Khan-Ghauri M.; Diévart P.; Catoire L.; Petersen E. L.; Mathieu O. Enhancing lithium-ion battery safety: Investigating the flame-retardant efficacy of bis(2,2,2-trifluoroethyl) carbonate during ethyl methyl carbonate combustion. Proc. Combust. Inst. 2024, 40 (1), 105559 10.1016/j.proci.2024.105559.

