# Supplementary material for: Bis(2,2,2-trifluoroethyl) Carbonate As a Fire Suppressant Candidate for Lithium-Ion Batteries
Source: Energy Fuels. 2025 Jan 22;39(10):4893–908. doi: 10.1021/acs.energyfuels.4c05359 (PMC11912212; doi:10.1021/acs.energyfuels.4c05359)
Supplement: Supplementary file 1 — ef4c05359_si_001.pdf [file ef4c05359_si_001.pdf]

Supplementary Information

Bis(2,2,2-trifluoroethyl) Carbonate as a Fire Suppressant Candidate for Lithium-Ion Batteries

Maryam Khan-Ghauri[1], Pascal Diévart[2,3], Claire M. Grégoire[1], Keisuke Kanayama[4], Yousef Almarzooq[1,5], Shintaro Takahashi[4], Takuya Tezuka[4], Hisashi Nakamura[4,], Laurent Catoire[3], Kaoru Maruta[4], Eric L. Petersen[1], and Olivier Mathieu[1,*]*

[1]*J. Mike Walker '66 Department of Mechanical Engineering, Texas A&M University, College Station, TX 77843, USA*

[2]*CNRS-INSIS, I.C.A.R.E., 1C, Avenue de la recherche scientifique, 45071 Orléans cedex 2, France*

[3] *Unité de Chimie et des Procédés (UCP), ENSTA Paris, Institut Polytechnique de Paris, Palaiseau, 91762, France*

[4] *Institute of Fluid Science, Tohoku University, Sendai, Miyagi 980-8577, Japan*

[5] *Mechanical Engineering Department, King Saud University, P.O. Box 800, Riyadh, 11421, Saudi Arabia*

Table S1: Rate constant coefficients of reactions involving $CH_2F_2/O_2/N_2$. In *italic* are the reaction rate coefficients used in Mathieu *et al.* (18), while the ones in **bold** correspond to the updated/added rate constants from Burgess *et al.* (39). $\left(k = AT^n exp\left(-E_a/RT\right)\right)$, units in cal, mol, and s. Note that the units from (39) are kJ, mol, and s, which were converted herein to cal, mol, and s for clarity.

Reactions (Updated)	A	n	E_a
$H_2 + F \leftrightarrows H + HF$	*2.56* $\times 10^{12}$	*0.5*	*649.8*
	**6.6** $\times \mathbf{10^{11}}$	**0.7**	**525.8**
$OH + F \leftrightarrows O + HF$	*2* $\times 10^{13}$	*0*	*0*
	**1.4** $\times \mathbf{10^{11}}$	**0.5**	**0**
$HO_2 + F \leftrightarrows O_2 + HF$	*2.89* $\times 10^{12}$	*0.5*	*0*
	**2.9** $\times \mathbf{10^{12}}$	**0.5**	**0**
$H_2O + F \leftrightarrows OH + HF$	*5.072* $\times 10^{7}$	*1.72*	*-1395.9*
	**1.3** $\times \mathbf{10^{8}}$	**1.6**	**-1099.4**
$CH_2F_2(+M) \leftrightarrows CHF + HF(+M)$	*2.34* $\times 10^{15}$	*0*	*82568*

LOW 5.33×10^{25} -8.635 87814<br>TROE 0.12 100 24000 467581761	**2.25×10^{15}**	**0**	**81809.7**
$CH_2F_2 + H \leftrightarrows CHF_2 + H_2$	*3.13×10^{4}*	*2.83*	*7392.4*
	**2.49×10^{15}**	**0**	**14947.1**
$CH_2F_2 + O \leftrightarrows CHF_2 + OH$	*1.2×10^{3}*	*3.17*	*5405.2*
	**1.86×10^{14}**	**0**	**13460.5**
$CH_2F_2 + OH \leftrightarrows CHF_2 + H_2O$	*1.32×10^{5}*	*2.35*	*1530.1*
	**1.68×10^{13}**	**0**	**7191.5**
$CHF_2 + H \leftrightarrows CHF + HF$	*1.49×10^{14}*	*-0.11*	*101.3*
	**6.5×10^{13}**	**0**	**0**
$CHF_2 + HO_2 \leftrightarrows CH_2F_2 + O_2$	*3×10^{12}*	*0*	*0*
	**1.3×10^{13}**	**0**	**5019**
$CHF + H_2O \leftrightarrows CH_2O + HF$	*5×10^{12}*	*0*	*6500.1*
	**5×10^{12}**	**0**	**6453**
$CHF + O_2 \leftrightarrows CHFO + O$	*2×10^{13}*	*0*	*16501.7*
	**3.4×10^{13}**	**0**	**7887**
$CHF + O \leftrightarrows CO + HF$	*8.43×10^{13}*	*0*	*0*
	**9×10^{13}**	**0**	**0**
$CHF + H \leftrightarrows CH + HF$	*6.4×10^{13}*	*0*	*0*
	**2×10^{14}**	**0**	**0**
$CF_2 + O_2 \leftrightarrows CF_2O + O$	*2.01×10^{13}*	*0*	*26503.3*
	**1.7×10^{13}**	**0**	**26529**
$CF_2 + OH \leftrightarrows CFO + HF$	*4×10^{12}*	*0*	*3499.5*
	**2×10^{12}**	**0**	**0**
$CF_2 + OH \leftrightarrows CF_2O + H$	*2×10^{13}*	*0*	*3499.5*
	**2.1×10^{11}**	**0.5**	**0**
$CF_2 + H \leftrightarrows CF + HF$	*3.98×10^{13}*	*0*	*4541*
	**3.6×10^{13}**	**0.5**	**4302**
$CF + O \leftrightarrows CO + F$	*4×10^{13}*	*0*	*999.6*
	**8×10^{13}**	**0**	**1410.1**
$CF + OH \leftrightarrows CO + HF$	*3×10^{13}*	*0*	*999.6*
	**8×10^{13}**	**0**	**1410.1**
$CHFO + OH \leftrightarrows CFO + H_2O$	*1.72×10^{9}*	*1.18*	*-447.1*
	**2.8×10^{13}**	**0**	**6620.3**
$CO + F + M \leftrightarrows CFO + M$	*3.09×10^{19}*	*-1.4*	*-488*
	**3×10^{18}**	**-1**	**30114**
$CH_2F_2 + CHF \leftrightarrows CHFCHF + HF$	*1×10^{13}*	*0*	*15001.4*
	**1.5×10^{13}**	**0**	**20076**
$CHFCF_2 + O \leftrightarrows CHF_2 + CFO$	*6×10^{9}*	*1*	*1150.6*
	**1.6×10^{13}**	**0**	**3991.3**
$CHF + CHF \leftrightarrows C_2HF + HF$	*1.7×10^{20}*	*-2.12*	*2380.7*
	**2.7×10^{13}**	**-2.12**	**-5019**

$CH_2F_2 + F \leftrightarrows CHF_2 + HF$	*9×10^{13}*	*0*	*1850.1*
	**5.54×10^{14}**	**0**	**2335**
Added Reactions	A	n	E_a
$CHF_2 + O_2 \rightarrow CF_2O + OH$	**1.5×10^{7}**	**1**	**0**
$CHF + F \rightarrow CF + HF$	**1×10^{13}**	**0**	**0**
$CHFCF_2 + O \leftrightarrows CHFO + CF_2$	**2.1×10^{13}**	**0**	**4015.2**
$CHFCF_2 + O \leftrightarrows CF_2O + CHF$	**1.2×10^{13}**	**0**	**3991.3**

Table S2: Rate constant coefficients for key reactions used in fluoromethane combustion studied by Sharma *et al.* (40). In *italic* are the reaction rate coefficients used in Mathieu *et al.* (18), while the ones in **bold** correspond to the added/updated/replaced rate constants from (40). $\left(k = AT^n exp\left(-E_a/RT\right)\right)$, units in cal, mol, and s. Note that the units from (40) are kJ, mol, and s, which were converted herein to cal, mol, and s for clarity.

Reactions (Added)	A	n	E_a
$H + CHFO \leftrightarrows HF + HCO$	**6.87**	**3.88**	**29756.2**
$O + CHFO \leftrightarrows FO + HCO$	**6.73×10^{6}**	**2.19**	**76912.1**
$O + CF_2O \leftrightarrows FO + CFO$	**4.52×10^{7}**	**2.02**	**80999.1**
$OH + CF_2O \leftrightarrows HOF + CFO$	**1.05×10^{7}**	**2.26**	**78680.8**
$FO + CHFO \leftrightarrows F_2O + HCO$	**2.36×10^{5}**	**2.6**	**82313.7**
$FO + CF_2O \leftrightarrows F_2O + CFO$	**1.30×10^{6}**	**2.44**	**86472.4**
$CH_3 + CHFO \leftrightarrows CH_3F + HCO$	**313**	**3.14**	**48398.7**
$CH_3 + CF_2O \leftrightarrows CH_3F + CFO$	**9770**	**2.88**	**48494.3**
$CH_2F + CHFO \leftrightarrows CH_2F_2 + HCO$	**27.8**	**3.32**	**44622.4**
$CH_2F + CF_2O \leftrightarrows CH_2F_2 + CFO$	**1470**	**3.01**	**47657.8**
$CHF_2 + CHFO \leftrightarrows CHF_3 + HCO$	**36.3**	**3.37**	**43833.7**
$F + CH_3F \leftrightarrows F_2 + CH_3$	**4.38×10^{6}**	**2.36**	**76816.5**
$F + CH_2F_2 \leftrightarrows F_2 + CH_2F$	**5.89×10^{7}**	**2.18**	**79828.0**
$F + CHF_3 \leftrightarrows F_2 + CHF_2$	**5.97×10^{8}**	**1.91**	**88527.8**
$F + CF_4 \leftrightarrows F_2 + CF_3$	**2.06×10^{9}**	**1.73**	**91634.9**
$O + CH_3F \leftrightarrows FO + CH_3$	**7.65×10^{5}**	**2.37**	**65415.9**
$O + CH_2F_2 \leftrightarrows FO + CH_2F$	**7.46×10^{6}**	**2.25**	**75812.7**
$O + CHF_3 \leftrightarrows FO + CHF_2$	**5.02×10^{7}**	**2.06**	**83628.2**
$O + CF_4 \leftrightarrows FO + CF_3$	**9.87×10^{7}**	**1.97**	**87667.4**
$OH + CH_3F \leftrightarrows HOF + CH_3$	**4.78×10^{4}**	**3.14**	**66610.9**
$OH + CH_2F_2 \leftrightarrows HOF + CH_2F$	**1.47×10^{6}**	**2.5**	**74163.6**
$OH + CHF_3 \leftrightarrows HOF + CHF_2$	**1.08×10^{7}**	**2.3**	**82385.4**
$OH + CF_4 \leftrightarrows HOF + CF_3$	**4.81×10^{7}**	**2.14**	**83484.8**
$FO + CH_3F \leftrightarrows F_2O + CH_3$	**2.52×10^{4}**	**2.84**	**74498.2**
$FO + CH_2F_2 \leftrightarrows F_2O + CH_2F$	**2.40×10^{5}**	**2.68**	**82050.8**
$FO + CHF_3 \leftrightarrows F_2O + CHF_2$	**1.74×10^{6}**	**2.48**	**89746.8**
$FO + CF_4 \leftrightarrows F_2O + CF_3$	**5.93×10^{6}**	**2.34**	**93905.5**

$CH_3 + CH_2F_2 \leftrightarrows CH_3F + CH_2F$	**440**	**3.07**	**46462.8**
$CH_3 + CHF_3 \leftrightarrows CH_3F + CHF_2$	**2320**	**2.92**	**51697.0**
$CH_3 + CF_4 \leftrightarrows CH_3F + CF_3$	$\mathbf{3.10 \times 10^4}$	**2.77**	**54158.8**
$CH_2F + CHF_3 \leftrightarrows CH_2F_2 + CHF_2$	**339**	**3.06**	**49569.8**
$CH_2F + CF_4 \leftrightarrows CH_2F_2 + CF_3$	**5050**	**2.9**	**52772.5**
$CHF_2 + CF_4 \leftrightarrows CHF_3 + CF_3$	**481**	**3.08**	**50382.5**
Reactions (Updated)	A	n	E_a
$CFO + H_2O_2 \leftrightarrows CHFO + HO_2$	*1* $\times 10^{11}$	*0*	*3900.9*
**$HO_2 + CHFO \leftrightarrows H_2O_2 + CFO$**	**205**	**4.16**	**15941.7**
$H + CH_3F \leftrightarrows HF + CH_3$	*2.75* $\times 10^{14}$	*0*	*31403.7*
	**13.6**	**3.72**	**22538.3**
$H + CH_2F_2 \leftrightarrows HF + CH_2F$	*5.5* $\times 10^{13}$	*0*	*34104.3*
	**3.26**	**3.94**	**27915.9**
$H + CHF_3 \leftrightarrows HF + CHF_2$	*8* $\times 10^{13}$	*0*	*40304.4*
	**1.92**	**4.04**	**32935.0**
$H + CF_4 \leftrightarrows HF + CF_3$	*1.1* $\times 10^{15}$	*0*	*44604.7*
	**2.58**	**4.04**	**35970.4**
Reactions (Replaced)	A	n	E_a
$H + CF_2O \leftrightarrows HF + CFO$	*5.5* $\times 10^{8}$	*1.42*	*18902.2*
DUP	*1.2* $\times 10^{10}$	*0.83*	*22302.3*
DUP	*2.4* $\times 10^{7}$	*1.88*	*35902.7*
**$H + CF_2O \leftrightarrows HF + CFO$**	**1.23**	**4.14**	**32241.9**

Table S3: Rate constant coefficients for reactions pertinent to hydrocarbon flame inhibition studied by Babushok *et al.* (41) that are key in BtFEC ($CF_3CH_2OCOOCH_2CF_3$ in the model) chemistry. In *italic* are the reaction rate coefficients used in Mathieu *et al.* (18), while the ones in **bold** correspond to the added/updated rate constants from (41). $\left(k = AT^n exp\left(-E_a/RT\right)\right)$, units in cal, mol, and s. Note that the units from (41) are kJ, mol, and s, which were converted herein to cal, mol, and s for clarity.

Reactions (Added)	A	n	E_a
$CF_3(+M) \leftrightarrows CF_2 + F(+M)$<br>LOW 5.0 $\times 10^{15}$ 0.0 249.62<br>H_2/2.00/ H_2O/6.00/ CH_4/2.00/ CO/1.50/ CO_2/2.00/ C_2H_6/3.00/ Ar/0.70/ CH_3F /6.00/ CH_2F_2 /6.00/ CHF_3 /6.00/ HF /2.00/	$\mathbf{1.0 \times 10^{15}}$	**0**	**82371**
$CF_3CF_2 \leftrightarrows CF_2 + CF_3$	$\mathbf{4.27 \times 10^{15}}$	**2.41**	**56240.5**
$CF_3CF_2 + F \leftrightarrows CF_3 + CF_3$	$\mathbf{3.16 \times 10^{13}}$	**0**	**0**
$CF_3O + CO \leftrightarrows CO_2 + CF_3$	$\mathbf{3.13 \times 10^{10}}$	**0**	**0**

$CFO + CF_2 \leftrightarrows CF_3 + CO$	**5.4×10^{11}**	**0**	**0**
$CF + CF \leftrightarrows C_2F_2$	**5×10^{13}**	**0**	**0**
$CF_2CF_2 + F \leftrightarrows CF_3CF_2$	**3.15×10^{13}**	**0**	**7770.1**
$F + F + M \leftrightarrows F_2 + M$	**1×10^{14}**	**0**	**0**
$F_2 + H \leftrightarrows F + HF$	**2.9×10^{9}**	**1.4**	**1328.9**
$CF_2 + F_2 \leftrightarrows CF_3 + F$	**1.2×10^{12}**	**0**	**2999.5**
$CFO + F_2 \leftrightarrows CF_2O + F$	**1×10^{12}**	**0**	**0**
$H_2 + F_2 \leftrightarrows HF + F + H$	**3.44×10^{12}**	**0**	**19789.7**
$F_2 + CO \leftrightarrows CFO + F$	**4.7×10^{11}**	**0**	**13499.1**
$C + F_2 \leftrightarrows CF + F$	**1.7×10^{12}**	**0**	**1500.9**
$CF + F_2 \leftrightarrows CF_2 + F$	**2.4×10^{12}**	**0**	**0**
$C_2H_4 + F_2 \leftrightarrows CH_2FCH_2 + F$	**4.8×10^{10}**	**0**	**4588.9**
$CH_4 + F_2 \leftrightarrows CH_3 + HF + F$	**2×10^{12}**	**0**	**11230.9**
$OH + F_2 \leftrightarrows HF + F + O$	**7×10^{13}**	**0**	**9000.9**
Reactions (Updated)	A	n	E_a
$CF_3COF + CF_3 \leftrightarrows CF_3CO + CF_4$	*2×10^{12}*	*0*	*9000.9*
	**2×10^{12}**	**0**	**9011**
$CF_3COF + CF_3CF_2 \leftrightarrows CF_3CO + CF_3CF_3$	*3×10^{11}*	*0*	*14000.9*
	**3×10^{11}**	**0**	**14006**
$CF_3CF_3 + CF_3 \leftrightarrows CF_4 + CF_3CF_2$	*3×10^{12}*	*0*	*11305*
	**3×10^{12}**	**0**	**11300.2**

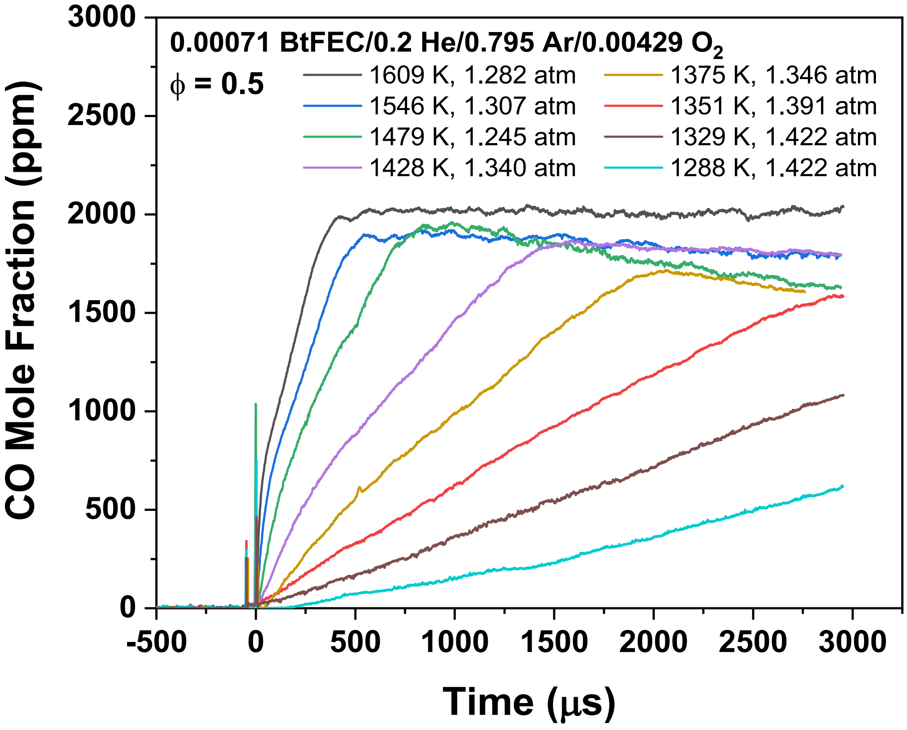

Figure S1: Experimental CO profiles from BtFEC oxidation with an equivalence ratio of Ø = 0.5

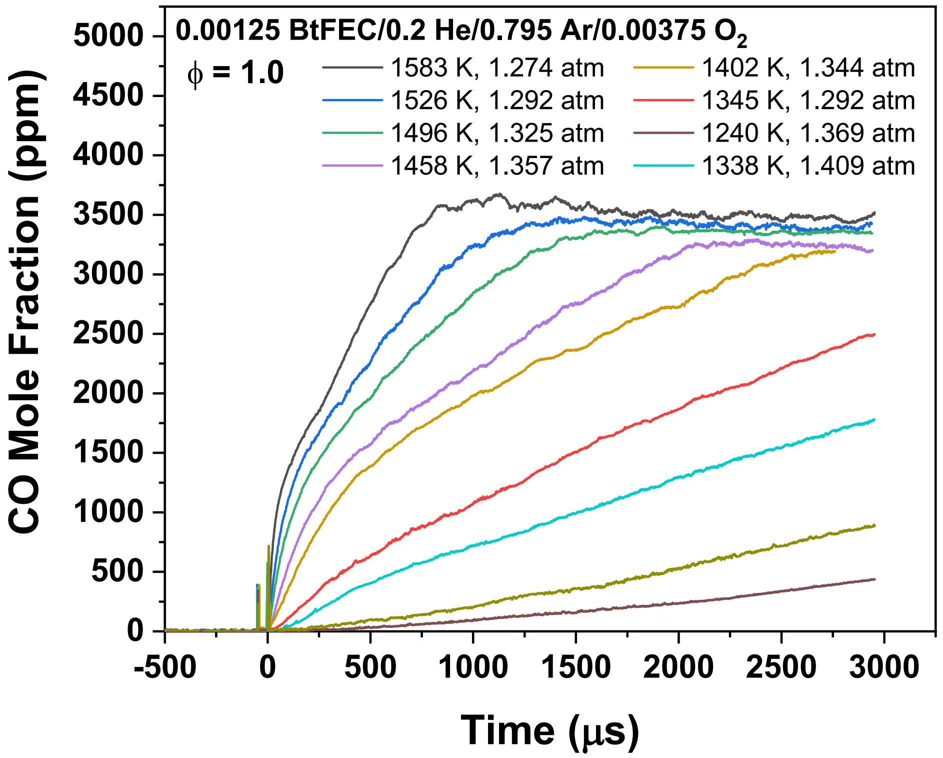

Figure S2: Experimental CO profiles from BtFEC oxidation with an equivalence ratio of Ø = 1.0

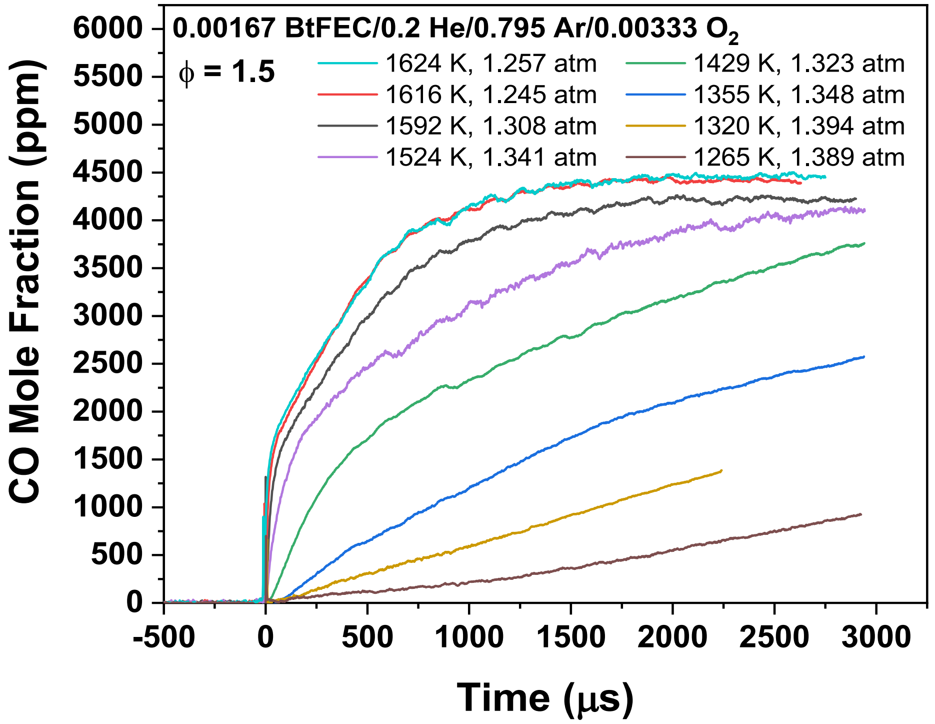

Figure S3: Experimental CO profiles from BtFEC oxidation with an equivalence ratio of Ø = 1.5

Table S4: Experimental OH* time-to-peak results for BtFEC/O_2/He/Ar mixture

**BtFEC / O_2 / He / Ar**								
**Φ = 0.5**			**Φ = 1.0**			**Φ = 1.5**		
**Temp (K)**	**Pressure (atm)**	**OH* Peak Time (μs)**	**Temp (K)**	**Pressure (atm)**	**OH* Peak Time (μs)**	**Temp (K)**	**Pressure (atm)**	**OH* Peak Time (μs)**
1609	1.28	320	1583	1.27	720	1624	1.26	510
1546	1.31	520	1526	1.29	915	1616	1.25	495
1479	1.25	800	1496	1.33	1264	1592	1.31	725
1428	1.34	1355	1458	1.36	1865	1524	1.34	1125
-	-	-	1402	1.34	2350	1429	1.32	2177

Table S5: Experimental OH* time-to-peak results for CH_4/O_2/Ar mixtures seeded with BtFEC

**CH_4 / O_2 / BtFEC / Ar**								
**Φ = 0.5**			**Φ = 1.0**			**Φ = 2.0**		
**Temp (K)**	**Pressure (atm)**	**OH* Peak Time (μs)**	**Temp (K)**	**Pressure (atm)**	**OH* Peak Time (μs)**	**Temp (K)**	**Pressure (atm)**	**OH* Peak Time (μs)**
1420	1.59	3128	1493	1.49	3014.7	1689	1.4	1645
1466	1.42	2201.5	1529	1.5	2411.2	1738	1.41	1219.2
1511	1.55	1495.7	1536	1.48	2343.8	1747	1.37	1114.8
1521	1.65	Weak signal	1556	1.46	1923.2	1753	1.28	566.3
1568	1.45	711.9	1571	1.41	1946.9	1779	1.3	480.3
1574	1.52	720.7	1601	1.4	1411.7	1841	1.38	275.2
1619	1.38	589.6	1632	1.42	1012.2	1901	1.36	247
1663	1.6	511.2	1642	1.45	909.8	1949	1.48	226.6
1691	1.31	392.4	1665	1.37	734	1955	1.31	160.3
1811	1.29	217.5	1709	1.36	517.2	2099	1.2	140.8
2022	1.31	104.7	1757	1.43	512.8	1689	1.4	1645

Table S6: Experimental OH* time-to-peak results for H_2/O_2/Ar mixtures seeded with BtFEC

**H_2 / O_2 / BtFEC / Ar**								
**Φ = 0.5**			**Φ = 1.0**			**Φ = 2.0**		
**Temp (K)**	**Pressure (atm)**	**OH* Peak Time (μs)**	**Temp (K)**	**Pressure (atm)**	**OH* Peak Time (μs)**	**Temp (K)**	**Pressure (atm)**	**OH* Peak Time (μs)**
1255	1.79	1326.4	1299	1.47	2469.1	1382	1.57	1645

1289	1.72	989.5	1328	1.55	1449.9	1419	1.5	1219.2
1315	1.68	910.5	1400	1.64	880.1	1425	1.69	1114.8
1342	1.62	639.9	1400	1.58	860.4	1507	1.52	566.3
1366	1.55	460.2	1403	1.46	737.9	1543	1.48	480.3
1395	1.62	Weak signal	1477	1.53	335.1	1634	1.52	275.2
1410	1.52	288.3	1543	1.58	250	1635	1.45	247
1514	1.57	131	1607	1.55	189.1	1685	1.41	226.6
1552	1.48	101.6	1616	1.42	165.2	1756	1.45	160.3
1649	1.48	58.4	1677	1.39	137.9	1813	1.46	140.8

Table S7: Experimental and computational Micro-flow reactor results in mole fractions for BtFEC

**BtFEC**	**Experiment**					**Computation**			
**Temp (K)**	**Φ = 0.5** Uncertainty (±7.611E-4)	**Φ = 1.0** Uncertainty (±7.244E-4)	**Φ = 1.5** Uncertainty (±7.428E-4)	**Pyrolysis** Uncertainty (±7.709E-4)	**Temp (K)**	**Φ = 0.5**	**Φ = 1.0**	**Φ = 1.5**	**Pyrolysis**
800	0.01447	0.01437	0.01486	0.01537	800	0.015	0.015	0.015	0.015
900	0.01473	0.01439	0.01482	0.01542	850	0.015	0.015	0.015	0.015
950	0.01522	0.01449	0.01442	0.01536	900	0.01499	0.01499	0.015	0.015
1000	0.0145	0.01235	0.01455	0.01538	950	0.01492	0.01495	0.01496	0.01498
1050	0.01407	0.01388	0.01392	0.01487	1000	0.01445	0.01463	0.01468	0.01482
1100	0.01054	0.01016	0.01155	0.01434	1050	0.01193	0.01263	0.01288	0.01359
1150	0.00486	0.00541	0.00565	0.01247	1100	0.00578	0.00674	0.00712	0.00806
1200	9.19946E-4	8.23027E-4	0.00215	0.00818	1150	5.79E-10	1.41E-5	3.80E-4	0.00131
1300	5.6404E-4	0.0014	0.00146	0.00292	1200	1.37E-15	0	4.61E-11	6.63E-5
					1250	1.23E-17	0	7.50E-18	3.39E-7
					1300	3.18E-21	4.61E-30	2.62E-21	4.54E-12

Table S8: Experimental and computational Micro-flow reactor results in mole fractions for CO

**CO**	**Experiment**					**Computation**			
**Temp (K)**	**Φ = 0.5** Uncertainty (±1.364E-3)	**Φ = 1.0** Uncertainty (±1.594E-3)	**Φ = 1.5** Uncertainty (±1.420E-3)	**Pyrolysis** Uncertainty (±7.831E-4)	**Temp (K)**	**Φ = 0.5**	**Φ = 1.0**	**Φ = 1.5**	**Pyrolysis**
800	8.49636E-4	0.00111	0.0013	2.34768E-4	800	3.82E-8	1.82E-8	1.23E-8	1.68E-9
900	9.89718E-4	0.00105	0.00129	5.18241E-4	850	9.20E-7	4.33E-7	3.65E-7	7.91E-8
950	0.00102	0.00126	3.40973E-4	3.21382E-4	900	1.13E-5	6.00E-6	4.66E-6	1.68E-6
1000	0.0011	0.00344	0.00137	5.26315E-4	950	8.71E-5	5.01E-5	3.96E-5	1.76E-5
1050	0.00237	0.00173	6.70246E-4	1.80145E-4	1000	7.39E-4	4.41E-4	3.57E-4	1.66E-4
1100	0.00688	0.00725	0.00447	1.89588E-4	1050	0.00585	0.00417	0.00359	0.00182
1150	0.01995	0.01785	0.01607	0.00195	1100	0.02149	0.01875	0.01761	0.01341
1200	0.02728	0.0306	0.02595	0.00888	1150	4.60E-4	0.02851	0.03613	0.03181

1300	4.04387E-4	0.03189	0.0284	0.01566	1200	1.99E-5	2.42E-4	0.02816	0.03416
					1250	6.27E-6	2.16E-4	0.02436	0.03331
					1300	3.34E-6	2.76E-4	0.02522	0.03224

Table S9: Experimental and computational Micro-flow reactor results in mole fractions for CO_2

CO_2	Experiment					Computation			
Temp (K)	Φ = 0.5 Uncertainty (±1.321E-3)	Φ = 1.0 Uncertainty (±1.321E-3)	Φ = 1.5 Uncertainty (±1.321-3)	Pyrolysis Uncertainty (±1.321E-3)	Temp (K)	Φ = 0.5	Φ = 1.0	Φ = 1.5	Pyrolysis
800	6.65994E-4	7.29756E-4	1.15196E-19	0.00101	800	2.61E-10	2.46E-10	2.47E-10	2.56E-10
900	1.91634E-4	2.9447E-4	3.03744E-5	6.99255E-4	850	6.46E-9	6.42E-9	6.31E-9	6.00E-9
950	0	-5.50571E-20	3.28068E-4	3.47178E-4	900	1.15E-7	1.13E-7	1.11E-7	1.10E-7
1000	2.15094E-4	9.40512E-4	1.2624E-4	1.92235E-4	950	2.09E-6	2.05E-6	2.04E-6	2.01E-6
1050	3.13579E-4	3.17756E-4	1.50089E-4	0.00157	1000	2.93E-5	2.79E-5	2.76E-5	2.69E-5
1100	0.00222	0.00242	0.00147	0.00145	1050	2.97E-4	2.47E-4	2.37E-4	2.04E-4
1150	0.00571	0.005	0.00464	0.00228	1100	0.00227	0.0017	0.00152	9.93E-4
1200	0.01312	0.01097	0.0073	0.00498	1150	0.04445	0.01629	0.00553	0.00249
1300	0.02641	0.00996	0.00781	0.00766	1200	0.04528	0.04977	0.0222	0.00372
					1250	0.04543	0.05074	0.02846	0.00438
					1300	0.04584	0.05158	0.02878	0.00488

Table S10: Experimental and computational Micro-flow reactor results in mole fractions for CF_2O

CF_2O	Experiment					Computation			
Temp (K)	Φ = 0.5 Uncertainty (±0.001)	Φ = 1.0 Uncertainty (±7.449E-4)	Φ = 1.5 Uncertainty (±5.731E-4)	Pyrolysis Uncertainty (±0.001)	Temp (K)	Φ = 0.5	Φ = 1.0	Φ = 1.5	Pyrolysis
800	1.98986E-4	2.58145E-5	1.30516E-4	3.64946E-4	800	3.07E-9	8.32E-10	3.78E-10	7.57E-16
900	2.39622E-4	1.98873E-4	3.27049E-5	3.98847E-4	850	9.99E-8	2.81E-8	2.01E-8	2.22E-15
950	8.03057E-5	1.93241E-4	3.08082E-4	3.60046E-4	900	1.53E-6	4.86E-7	2.63E-7	4.60E-13
1000	2.3175E-4	4.04753E-4	4.20598E-5	4.61193E-4	950	1.43E-5	4.99E-6	2.73E-6	4.81E-11
1050	7.72285E-4	3.37274E-4	3.06396E-4	5.61257E-4	1000	1.49E-4	6.41E-5	4.05E-5	2.53E-9
1100	0.00342	0.00251	6.53703E-4	2.20046E-4	1050	0.00139	8.28E-4	6.26E-4	5.19E-8
1150	0.01059	0.00787	0.00588	5.13856E-5	1100	0.00598	0.00421	0.0034	5.83E-7
1200	0.01925	0.0149	0.01091	3.68297E-4	1150	0.01795	0.01095	0.0075	3.36E-6
1300	0.02206	0.01302	0.01146	0	1200	0.01833	0.0137	0.00943	1.11E-5
					1250	0.01848	0.01372	0.00911	2.01E-5
					1300	0.01895	0.01381	0.00883	2.93E-5

Table S11: Experimental and computational Micro-flow reactor results in mole fractions for CHF_3

CHF_3	Experiment					Computation			
Temp (K)	Φ = 0.5 Uncertainty (±1.494E-4)	Φ = 1.0 Uncertainty (±1.697E-4)	Φ = 1.5 Uncertainty (±1.984E-4)	Pyrolysis Uncertainty (±2.866E-4)	Temp (K)	Φ = 0.5	Φ = 1.0	Φ = 1.5	Pyrolysis
800	1.85422E-4	1.92382E-4	2.5072E-4	2.12084E-4	800	3.46E-8	1.77E-8	1.22E-8	1.87E-9
900	1.93566E-4	2.4248E-4	2.65813E-4	1.84415E-4	850	7.35E-7	3.88E-7	3.39E-7	8.382E-8
950	2.477E-4	1.77084E-4	1.77084E-4	1.77084E-4	900	7.68E-6	4.83E-6	4.01E-6	1.69E-6
1000	2.60237E-4	3.76708E-4	2.48005E-4	2.20506E-4	950	4.98E-5	3.57E-5	3.10E-5	1.67E-5
1050	4.34916E-4	4.02882E-4	3.49562E-4	2.07299E-4	1000	3.16E-4	2.42E-4	2.18E-4	1.31E-4
1100	0.00123	0.00159	0.00114	3.0466E-4	1050	0.00184	0.00154	0.00144	0.00104
1150	0.00299	0.00326	0.00345	0.00109	1100	0.00571	0.00595	0.00609	0.00634
1200	0.00167	0.00273	0.00397	0.00345	1150	3.36E-5	0.00484	0.00992	0.01306
1300	0	0.00339	0.00366	0.00573	1200	3.45E-7	5.41E-8	9.70E-4	0.01033
					1250	1.38E-7	3.71E-8	1.25E-5	0.00615
					1300	4.38E-7	4.39E-8	2.77E-5	0.00285

Table S12: Experimental and computational Micro-flow reactor results in mole fractions for HF (normalized)

HF	Experiment					Computation (Normalized)			
Temp (K)	Φ = 0.5 Uncertainty (±0.05)	Φ = 1.0 Uncertainty (±0.05)	Φ = 1.5 Uncertainty (±0.05)	Pyrolysis Uncertainty (±0.05)	Temp (K)	Φ = 0.5	Φ = 1.0	Φ = 1.5	Pyrolysis
800	0.01037	0.00805	0.00256	0.02548	800	1.39E-7	3.52E-8	2.29E-8	2.02E-8
900	0.01242	0.01879	0.00721	0.03555	850	4.06E-6	9.21E-7	5.81E-7	2.84E-7
950	0	0.0023	0.00352	0.01791	900	6.08E-5	1.44E-5	8.48E-6	3.24E-6
1000	0.02048	0.1627	0.01098	0.02309	950	5.65E-4	1.45E-4	8.58E-5	3.818E-5
1050	0.02399	0.02718	0.02466	8.7435E-4	1000	0.00564	0.00166	0.00104	4.23E-4
1100	0.12462	0.1867	0.09299	0.01017	1050	0.05102	0.02028	0.01479	0.00388
1150	0.37651	0.42707	0.41743	0.07095	1100	0.21261	0.09953	0.07749	0.03533
1200	0.73961	1	0.81215	0.35406	1150	0.86393	0.41244	0.23905	0.2285
1300	1	0.97508	1	1	1200	0.91358	0.84334	0.73844	0.56658
					1250	0.94739	0.9235	0.92249	0.81853
					1300	1	1	1	1

Table S13: Experimental and computational Micro-flow reactor carbon balance

CB	Experiment					Computation			
**Temp (K)**	**Φ = 0.5**	**Φ = 1.0**	**Φ = 1.5**	**Pyrolysis**	**Temp (K)**	**Φ = 0.5**	**Φ = 1.0**	**Φ = 1.5**	**Pyrolysis**
800	0.99261	0.98527	1.01467	1.05109	800	1	1	1	1
900	1.00498	0.99485	1.0109	1.05196	850	0.99996	0.99998	0.99998	1
950	1.04064	0.98913	0.97866	1.04018	900	0.99954	0.99974	0.99979	0.99991
1000	0.99479	0.90547	0.99409	1.04523	950	0.99684	0.99801	0.99836	0.99913
1050	1.00104	0.96742	0.94911	1.0281	1000	0.98121	0.98688	0.98872	0.99287
1100	0.9053	0.88484	0.88556	0.98691	1050	0.93167	0.94146	0.94533	0.95197
1150	0.91336	0.86714	0.82745	0.91357	1100	0.90167	0.89481	0.89126	0.84073
1200	0.95337	0.91519	0.85085	0.8186	1150	0.92232	0.90195	0.88961	0.77289
1300	0.7364	0.94021	0.85268	0.63926	1200	0.92714	0.91507	0.88927	0.70561
					1250	0.92642	0.91459	0.88897	0.64255
					1300	0.92838	0.9144	0.88957	0.58588

Table S14: Micro-flow reactor wall temperature profiles for temperature 800 K – 1050 K.

Location (cm)	Temp (K) - 800 K	Location (cm)	Temp (K) - 900 K	Location (cm)	Temp (K) - 950 K	Location (cm)	Temp (K) - 1000 K	Location (cm)	Temp (K) - 1050 K
2.44738	406.0241	2.48838	432.53012	2.33542	425.3012	2.48865	463.85542	2.3459	459.03614
2.94737	463.85542	2.98847	502.40964	2.84572	497.59036	2.99909	553.01205	2.84621	555.42169
3.45791	565.06024	3.49924	630.12048	3.35638	613.25301	3.49995	714.45783	3.34692	700
3.94831	695.18072	4.00002	781.92771	3.84723	796.38554	3.99078	895.18072	3.85808	873.49398
4.44848	774.6988	4.49009	873.49398	4.3476	900	4.50121	981.92771	4.3586	993.9759
4.94819	801.20482	4.99996	895.18072	4.8474	936.14458	4.79697	998.79518	4.83815	1046.98795
5.44752	781.92771	5.50946	873.49398	4.95957	940.96386	5.00088	1003.61446	5.00127	1049.39759
5.94644	714.45783	5.99814	801.20482	5.35696	921.68675	5.50008	969.87952	5.34773	1034.93976
6.44501	606.0241	6.49649	666.26506	5.84571	856.62651	5.99882	880.72289	5.84665	967.46988
6.95352	468.6747	6.99458	502.40964	6.34413	731.3253	6.49689	714.45783	6.34479	808.43373
7.4523	384.33735	7.49323	403.61446	6.84217	560.24096	6.99479	526.50602	6.84272	625.3012
				7.35075	430.12048	7.50369	434.93976	7.34086	466.26506
								7.83974	393.9759

Table S15: Micro-flow reactor wall temperature profiles for temperature 1100 K – 1300 K.

Location (cm)	Temp (K) - 1100 K	Location (cm)	Temp (K) - 1150 K	Location (cm)	Temp (K) - 1200 K	Location (cm)	Temp (K) - 1300 K
2.10104	434.93976	1.79487	391.56627	2.08068	437.3494	2.09089	439.75904
2.60131	526.50602	2.28492	480.72289	2.57085	540.96386	2.59138	557.83133
3.09147	627.71084	2.78539	596.38554	3.0815	654.21687	3.09188	675.90361
3.59262	822.89157	3.29641	753.01205	3.59311	880.72289	3.59321	892.77108
4.11399	998.79518	3.78736	948.19277	4.08408	1078.31325	4.09473	1131.3253
4.61415	1078.31325	4.30849	1095.18072	4.60478	1174.6988	4.60564	1275.90361
4.89972	1095.18072	4.80847	1153.01205	5.11467	1198.79518	4.90146	1300
5.11381	1097.59036	4.97157	1153.01205	5.61377	1153.01205	4.99322	1302.40964
5.59256	1056.62651	5.30786	1140.96386	6.10202	1030.12048	5.10536	1302.40964
6.09113	948.19277	5.79648	1061.44578	6.58933	796.38554	5.60452	1263.85542
6.60929	745.78313	6.30483	904.81928	7.0871	593.9759	6.10294	1138.55422
7.09695	553.01205	6.80242	680.72289	7.60584	459.03614	6.6106	900
7.59544	434.93976	7.31069	514.45783	8.10469	384.33735	7.09754	622.89157
8.10463	377.10843	7.80936	418.07229			7.606	478.31325
						8.09464	401.20482

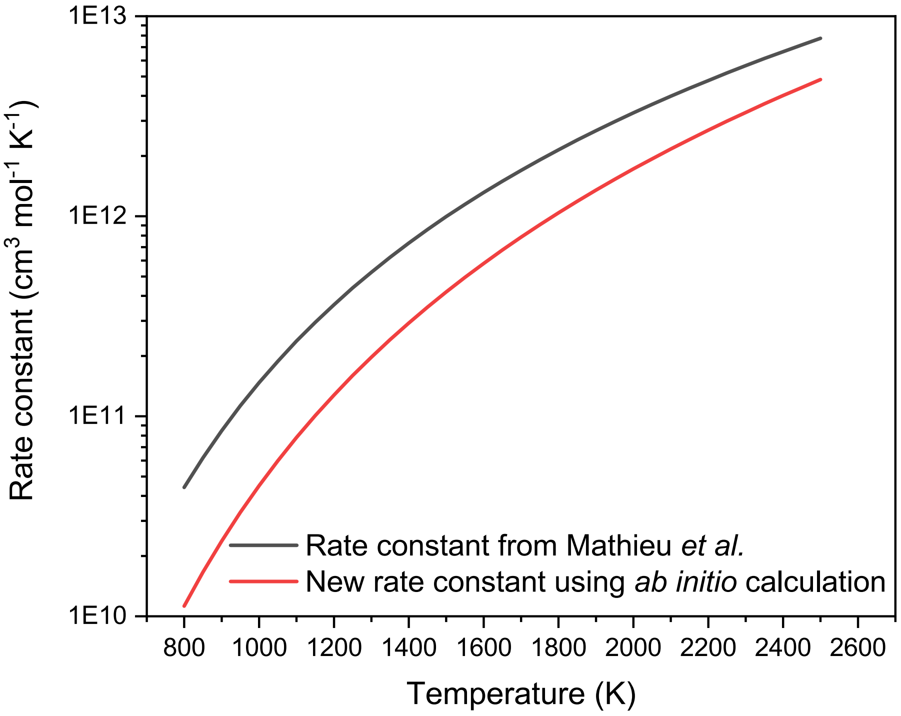

Figure S4: Rate constant comparison between rate constants used in previous model by Mathieu *et al.* referenced in text, and updated rate coefficients calculated in this study for the reaction CF_3 + CH_2O ⇆ HCO + CHF_3.

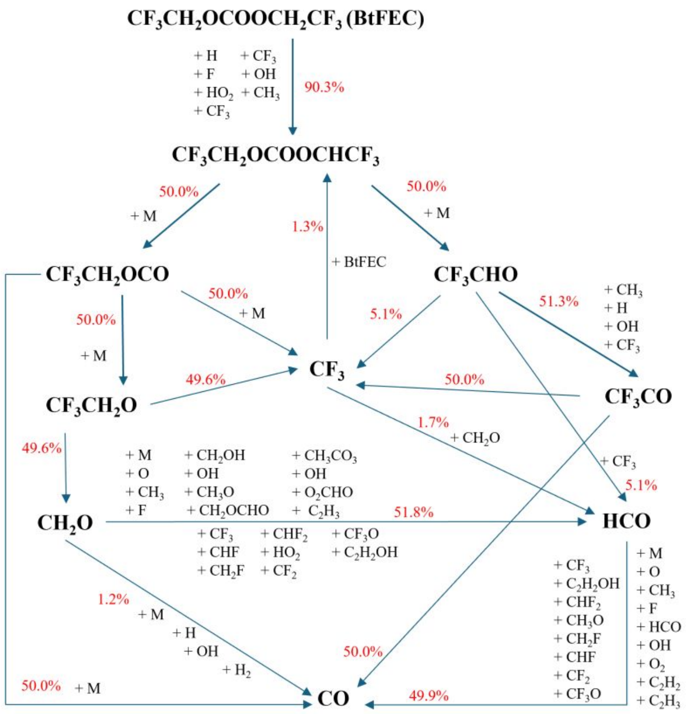

Figure S5: Reaction pathway from BtFEC to CO with relative rate of production percentages for the produced species for φ = 0.5, 1050 K, 1.34 atm. No species added on the arrow indicates a decomposition reaction. The percentage represents the contribution of reactions of the upper species to form the lower species.
